# Pulchinenoside B4 ameliorates oral ulcers in rats by modulating gut microbiota and metabolites

**DOI:** 10.1007/s00253-024-13099-1

**Published:** 2024-04-09

**Authors:** Dewei Luo, Li Yan, Zhujun Wang, Xiaofan Ji, Na Pei, Jing Jia, Yingying Luo, Hui Ouyang, Shilin Yang, Yulin Feng

**Affiliations:** 1 Jiangxi University of Traditional Chinese Medicine, No. 818 Yunwan Road, Nanchang, 330002 People’s Republic of China; 2 National Pharmaceutical Engineering Center for Solid Preparation in Chinese Herbal Medicine, No. 56 Yangming Road, Nanchang, 330006 People’s Republic of China; 3 Research Center of Natural Resources of Chinese Medicinal Materials and Ethnic Medicine, Jiangxi University of Traditional Chinese Medicine, No. 818 Yunwan Road, Nanchang, 338004 People’s Republic of China; 4 Xinyu University, No. 2666 Yangguang Road, Xinyu, 338004 People’s Republic of China

**Keywords:** Pulchinenoside B4, Oral ulcer, Metabolomics, Fecal microbiome

## Abstract

**Abstract:**

Pulchinenoside B4, a natural saponin monomer from the *Pulsatilla* plant, plays an important role as an immunomodulator in the treatment of acute inflammation. Oral ulcer (OU) is a common ulcerative injury disease that occurs in the oral mucosa, including mucosal ulceration and abnormalities of lips and tongue. A close correlation exists between gut microbiota and circulating metabolites in patients with OU. However, the correlation between gut microbiota and serum metabolomics is not clear. Therefore, this study aimed to explore the changes in gut microbiota and metabolites in OU. The 16S ribosomal RNA (*16S rRNA*) gene sequencing was used to detect the changes in the composition of gut microbiota in OU rat model. Moreover, the endogenous small metabolites were explored by collecting the non-targeted serum metabolomics data. A total of 34 OU-related biomarkers were identified, mainly related to fatty acid metabolism and inflammatory pathways. The administration of B4 effectively reduced the occurrence of OU and restored the levels of multiple endogenous biomarkers and key gut microbial species to the normal level. This study demonstrated that the gut microbiota and metabolites were altered in the OU rat model, which were significantly restored to the normal level by B4, thereby showing good application prospects in the treatment of OU.

**Key points:**

• *The first investigating the correlation between OU and gut microbiota.*

• *A close correlation between metabolites and gut microbiota in OU disease was successfully identified.*

• *Pulchinenoside B4 ameliorates oral ulcers in rats by modulating gut microbiota and metabolites.*

**Supplementary Information:**

The online version contains supplementary material available at 10.1007/s00253-024-13099-1.

## Introduction

The incidence of oral ulcer (OU), one of the most common oral mucosal diseases, is increasing year by year, affecting up to 30% of the population (Toche et al. 2007). Its causes are still unclear, and no specific treatment is available for this disease. The frequent recurrence and severe pain caused by OU seriously affect the patient’s quality of life. It also leads to eating disorders, thus affecting the patients’ mental and psychological conditions (Yogarajah and Setterfield 2021; Cabras et al. 2020). Recently, researchers focused on the etiology and immune factors associated to OU (Jin et al. 2020). Studies indicated that the abnormal immune function is an important cause of OU (Chen et al. 2020a, b). OU is a damage of oral epithelial cells mediated by T lymphocytes during an immune response, demonstrating the correlation between T lymphocytes and OU (Bank et al. 2003). OU is manifested as a non-specific inflammatory reaction, including cell edema, dissolution, ulceration, or necrosis, leading to the formation of ulcers (Minhas et al. 2019; Poulter and Lehner 1989). Macrophages are involved in tissue remodeling and damage repair by the rearrangement of the metabolic state and function, regulation of inflammation, and maintenance of the homeostasis (Surboyo et al. 2020). The expression of the surface marker of M1-type macrophages cluster of differentiation 68 (CD 68) in OU tissue reflects the immune status of the OU microenvironment to a certain extent (AI-Talqani et al. 2018).

The *Pulsatilla* plant has been widely used in China in the treatment of bacterial diseases for hundreds of years (Li et al. 2019). *Pulsatilla* saponin B4 is the most abundant monomer in *Pulsatilla*, thus attracting the attention of researchers due to its good therapeutic potential. Its pharmacological activities also make it an important quality control (QC) component. Moreover, it possesses anti-tumor, neuroprotective, and anti-angiogenetic activities (Li et al. 2019; Kang et al. 2019).

The human intestine contains many different microorganisms (10^13^–10^14^) known as gut microbiota, whose number is 10 times greater than the total number of human cells (Lee et al. 2021). The normal physiological functions of the human body is maintained by the gut microbiota and their abundant coding genes. The human body is nowadays considered as a superorganism composed of eukaryotic cells and a symbiotic microbial community, and researchers have recently focused on this closely related biological coexistence (Dethlefsen et al. 2007). Numerous studies on gut microbiota showed that pathological conditions easily cause an imbalance of the intestinal microenvironment and gut microbiota. This imbalance is closely related to several diseases, such as diarrhea, hypertension, alcoholic liver injury, colorectal cancer, and gallstones (Simon and Gorbach 1984). However, studies exploring the correlation between gut microbiota and related metabolites in OU are limited. The administration of a rectal suppository improves the bioavailability and sustained release of the drug while avoiding the side effects related to oral administration, such as the first-pass effect and degradation of the drug by gastric enzymes (Bergogne and Bryskier 1999). Numerous studies showed the amelioration of the pathogenesis of immune diseases after suppository administration (Zeng et al. 2022; Sun et al. 2018). The rectal administration also changes the composition of gut microbiota, leading to the improvement of microbial metabolism, consequently improving the disease (Foppoli et al. 2019; Seoane-Viaño et al. 2021).

The current study aimed to determine the direct correlation between blood metabolites and gut microbiota to explore the mechanism characterizing OU pathogenesis. The *16S rRNA* gene sequencing technology was used to assess the changes in the composition of gut microbiota. Moreover, OU biomarkers were analyzed using non-targeted serum metabolomics.

## Materials and methods

### Instruments and reagents

The following systems and instruments were used in this study: Triple TOF 5600^+^ system (AB SCIEX, Framingham, MA, USA), Analyst TF1.6 and Peakview 1.2 data processing system (AB SCIEX, Framingham, MA, USA), Markerview 1.2 data processing system (AB SCIEX, Framingham, MA, USA), Simca-p 14.1 (Umetrics, Umea, Sweden), Milli-q ultrapure water system (Millipore, Burlington, MA, USA), Centrifuge 5810R refrigerated centrifuge (Eppendorf, Hamburg, Germany), ACQUITY UPLC® H-Class ultra-high performance liquid chromatograph (Waters, Milford, MA, USA), anesthesia machine (Reward, R580, RWD Life Science, San Diego, CA, USA), pathology gross camera (OPLENIC, SY-12, Oplenic Optronics Equipment Co., Ltd, Zhejiang, China), hemocytometer (SYSMEX, XT-20001, Sysmex, Kobe, Japan), flow cytometer (Gallios, Beckman, Pasadena, CA, USA), electronic balance (Beijing Sartorius Scientific Instruments Co., Ltd., Beijing, China), GeneAmp® 9700 polymerase chain reaction (PCR) (American Applied Biosystems, Waltham, MA, USA), QuantiFluor™-ST Blue Fluorescence Quantitative System (Promega, Madison, WI, USA), and manual rotary microtome (Leica, RM2235, Leica, Wetzlar, Germany).

The following reagents and chemicals were used in this study: methanol, acetonitrile (chromatographically pure grade, Fisher Scientific, Fairlawn, NJ, USA), formic acid (chromatographically pure, Sigma-Aldrich Co. Ltd, St Louis, MO, USA), distilled water (Hangzhou Wahaha Group Co., Ltd., Hangzhou, China), glacial acetic acid (Western Long Science Co., Ltd., Shenzhen, China, batch number 180510), chloral hydrate (Tianjin Damao Chemical Reagent Factory, Tianjin, China, batch number: 20180518), isoflurane (Shenzhen Reward Life Technology Co., Ltd., Shenzhen, China, 217,180,501), phosphate-buffered saline (PBS) buffer dry powder (Coolaber company, Beijing, China, PM30143050000), 0.01 M sodium citrate buffer (Solarbio company, Beijing, China, 1114A0144), 3,3′-diaminobenzidine kit (Kangwei Century, Jiangsu, China, 35,620), xylene (Tianjin Hengxing Chemical Reagent Manufacturing Co., Ltd., Tianjin, China, 20,210,520), absolute ethanol (Xilong Science, Guangdong, China, 2,105,299), anti-CD68 antibody (Abcam, Zhejiang, China, GR3326063-11), primary antibody dilution working solution (5% goat serum, BOSTER, Wuhan, China, 15L08A09) + 0.5% bovine serum albumin (GENVIEW, Florida, USA, 0703010160) + 0.3% Triton X-100 (Solarbio company, Beijing, China, 829I0213), hematoxylin (Solarbio company, Beijing, China, 20,200,629), neutral gum (China National Pharmaceutical Group Chemical Reagent Co., Ltd., Shanghai, China, 20,190,411), and immunohistochemistry (IHC) pen (Biosharp, Anhui, China).

The reference chemicals included 2-chloro-L-phenylalanine alanine, 4-hydroxybenzophenone, lathosterol, 1,2-dichloroethane, *N*-arachidonoyl tyrosine, stearoylglycine, and L-tyrosine. All the chemicals had a standard purity of > 98% and were purchased from Shanghai Yuanye Bio Technology Co., Ltd (China).

Pulchinenoside B4 suppositories were provided by Dr. Guosong Zhang (Jiangxi University of Chinese Medicine, Jiangxi, China) and included 28 mg/tablet, 14 mg/tablet, 7 mg/tablet, and 3.5 mg/tablet. The blank suppositories (except for the absence of pulchinenoside B4, all other excipients are the same as those in B4 suppositories) were also provided (batch number: 20200715).

The kits used in this study were the following: E.Z.N.A.Stool DNA kit (Omega Bio-Tek, Norcross, GA, USA), AxyPrepDNA gel recovery kit (AXYGEN, Tewksbury, MA, USA), TruSeqTM DNA Sample Prep Kit (Illumina, San Diego, CA, USA), TransStart Fastpfu DNA Polymerase (TransGen Biotech Co., Ltd, Beijing, China), and TRIzol (Thermo Fisher Technology Co., Ltd., Waltham, MA, USA).

### Compound source

B4 suppository was purchased from Jiangxi Materia Medica Co., Ltd., (Nanchang, China) with a purity > 98% (batch number 20181107). The structural formula of *Pulsatilla* saponin B4 is shown in Supplementary Fig. S1.

### Animals

Forty specific-pathogen-free male Sprague Dawley rats, weighing 200–220 g, were purchased from the Hunan Slack Jingda Experimental Animal Co., Ltd. (Changsha, China) [license number SCXK (Hua)]. Rats were housed in a controlled environment with a temperature of 24–26 °C, suitable humidity, 12/12 h light and dark conditions, and food and water ad libitum and subjected to 1 week of acclimatization before starting the experiments. All animal experiments were performed according to the guidelines of the National Institutes of Health and approved by the Ethics Committee of Jiangxi University of Chinese Medicine.

### Establishment of OU rat model

Rats were treated with an intraperitoneal injection of fluorouracil at a dose of 40 mg/kg on the first and third day. Then, the rats were anesthetized by an intraperitoneal injection of 10% chloral hydrate solution at 24 h after the last injection. Then, a 3 mm × 3 mm filter paper impregnated with 100% glacial acetic acid was placed closely adhering to the right buccal mucosa of the rat for 60 s. A round or oval white lesion appeared on the buccal mucosa in this area, indicating an injury.

The successfully establishment of OU was based on the area of ulcer and the OU rats were randomly divided into 4 groups (8 rats in each group): model group (Model, which only received vehicle treatment), B4-low group (B4-low, which received B4 treatment at a dose of 30 mg/kg each time), B4-medium group (B4-medium, which received B4 treatment at a dose of 60 mg/kg each time), and B4-high group (B4-high, which received B4 treatment at a dose of 120 mg/kg each time). The control group (Control) was also set with 8 rats treated only with saline. After 24 h of the damage of the mucosa with glacial acetic acid, the rats in the B4 group were treated with the same concentration as above of B4 suppository by rectal administration (twice a day for 5 days, 30, 60, and 120 mg/kg dose for each time), while rats in the control group and model group were treated with suppository containing only vehicle. Plasma and cecal contents were collected 24 h after the last administration.

### Collection and processing of plasma samples

Rats were anesthetized by an intraperitoneal injection of 10% chloral hydrate solution 24 h after the last administration. Blood samples were collected from the abdominal aorta and placed into a 10-mL Eppendorf (EP) Micro Test Tube after heparin sodium treatment and then centrifuged at 4 °C and 4500 rpm for 5 min. The supernatant was collected, aliquoted in EP tubes, and stored at − 80 °C for subsequent use.

A total of 5.03 mg 2-chloro-L-phenylalanine was accurately weighed into a 25-mL volumetric flask, and 25 mL methanol was added to dilute it 20 times, preparing a working solution at a concentration of 10.06 μg/mL, which was then stored at 4 °C. Fifty microliters of rat plasma was taken, in which 200 μL of the previously prepared working solution was added and mixed for 30 s using a vortex. The mixture was left for 10 min, centrifuged at 12,000 rpm and 4 °C for 15 min, then the supernatant was collected and stored at − 20 °C. The quality control samples were prepared by taking an equal amount of plasma from each sample. A 100 μL QC plasma was collected and an amount four times the amount of internal standard working solution was added. The mixing, centrifugation, and storage were performed similarly to that of the other samples.

### Measurement of rat OU area and hematoxylin and eosin (H&E) staining

The OU area of the rats in the model and each administration group was measured using a vernier caliper, calculated according to Eq. (1) and the average value was calculated:

1 $$OU\;area\;({mm}^2)=\pi\times d1\times d2\times1/4$$

where π = 3.14, and d1 and d2 were the largest transverse and longitudinal diameters of OU, respectively.

The rats in each group were sacrificed by cervical dislocation. The OU tissues were cut, collected, fixed in 10% formaldehyde solution for 24 h, dehydrated, paraffinized, and sectioned. Pathological changes in the OU tissues were observed on H&E sections stained using a standard protocol. The standard protocol is described in the Supplementary Material.

### Determination of CD68 expression in rat OU tissue sections using IHC

#### Sample preparation

Fresh rat OU tissues were fixed in 10% neutral formalin, paraffinized, cut into 8 μm-thick sections, and subjected to H&E, IHC, and other immunostainings.

#### Dewaxing of paraffinized tissue sections

Dewaxing of paraffinized tissue sections was performed at 60 °C for 1 h, followed by xylene for 5 min (two times). The tissues were successively dehydrated with gradient ethanol (anhydrous ethanol, 95%, 85%, and 75%) and then hydrated in distilled water for 5 min.

#### Antigen retrieval

Sodium citrate buffer solution (pH 6.0) in a pressure cooker was used for antigen retrieval. Tissue sections were immersed in the repair solution in the pressure cooker and uniformly heated. The heat was released after 1–2 min, and the pressure cooker was left to cool until reaching room temperature. Tissue sections were taken out and rinsed in distilled water, and then phosphate buffer saline (PBS) three times (5 min each time).

#### Background blocking and target detection

Tissues were fixed using an immunohistochemical pen circle. Then, endogenous peroxidase blocking solution was added dropwise to the sections and they were incubated at room temperature for 10 min and then rinsed with PBS 5 times (5 min each time). Next, the normal goat serum working solution was added dropwise and the sections were incubated at room temperature. Sections were then spin-dried after 10 min, treated with the primary antibody (1:500 diluted CD68 antibody), incubated at 4 °C overnight, and rinsed with PBS 5 times (5 min each time). The sections were then incubated with the biotin-labeled goat anti-rabbit/mouse secondary antibody working solution for 10 min, rinsed with PBS 5 times (5 min each time), incubated for 10 min with horseradish peroxidase-labeled streptavidin at room temperature, and rinsed with PBS for 5 times (5 min each time).

#### Color development

3,3′-Diaminobenzidine (DAB) kit working solutions A and B (solutions A and B are the ready-to-use solutions in the kit, and the concentrations are not indicated) were mixed with a volume ratio of 1:19 at 4 °C in the dark (effective for 1 h). The prepared DAB solution was added to the sections and color development was observed under the microscope to evaluate the best effect. The sections were then rinsed with tap water to terminate the color development, counterstained with hematoxylin for 4 min, and washed with water. The sections were dehydrated with gradient ethanol (75%, 85%, and 95%-anhydrous ethanol) for 4 min, cleared with xylene for 5 min × 3, sealed with neutral gum, dried, and observed under an optical microscope.

#### Interpretation of CD68 IHC staining results

CD68 IHC in the oral mucosal tissue sections was observed under an optical microscope. Cells positive for CD68 showed specific brown-yellow particles in the cytoplasm. Images were taken and ImageJ software (Baocheng Biology company, Hangzhou, China) was used to measure the positive stained area.

### Detection conditions and optimization of liquid chromatography-mass spectrometry

#### Liquid chromatography conditions

Different mobile phase compositions were used, such as water-acetonitrile, 0.1% formic acid water-acetonitrile, and 5 mmol ammonium formate water-acetonitrile (Waters ACQUTITY UPLCTMHSS T3 chromatographic column, Waters, Milford, MA, USA), and different injection volumes were used, such as 1 μL, 3 μL, and 5 μL. The combination of 0.1% formic acid water (solution A) and acetonitrile (solution B) was selected as the mobile phase, and the ACQUTITY UPLCTMHSS T3 chromatographic column was selected as a separation column with 3-μL injection volume to comprehensively obtain a better peak shape and peak intensity as well as considering the effects of continuous long-time injection on the stability of the instrument and chromatographic column. The gradient elution scheme was as follows: 0–0.01 min, solution B maintained at 5%; 0.01–3 min, solution B increased from 5 to 20%; 3–5 min, solution B increased from 20 to 45%; 5–7 min, solution B increased from 45 to 55%; 7–8 min, solution B increased from 55 to 60%; 8–15 min, solution B increased from 60 to 65%; 15–17 min, solution B increased from 65 to 70%; 17–20 min, solution B increased from 70 to 80%; 20–23 min, solution B increased from 80 to 95%; 23–23.1 min, solution B decreased from 95 to 5%; and 23.1–26 min, solution B maintained at 5%.

#### Mass spectrometry conditions

(1) Electrospray ionization was used in positive ion mode, and the mass scanning range was 50–1250 Da. The other conditions were as follows: spray voltage (ISVF), 5500 V; atomizer temperature (TEM), 500 °C; collision energy (CE), 35 eV; CE superimposition, 35 ± 15; curtain air (CUR), 30 psi; atomization gas (GS), 50 psi; auxiliary gas, 50 psi; declustering voltage (DP), 100 V; and data acquisition time, 26 min. Moreover, the product ion (TOF–MS-IDA-MS/MS) scanning method starting from the parent ion was adopted, and the second level was triggered using multiple mass defects (MMDF) and dynamic background subtraction (DBS).

#### Level data collection

(2) Electrospray ionization was used in negative ion mode, and m/z of 50–1250 was selected as the mass scanning range. The other conditions were as follows: ISVF, − 4500 V; TEM 500 °C; CE-35 eV, collision CES, 35 ± 15; CUR, 30 psi; GS2, 50 psi; GS2, 50 psi; DP, − 100 V; and data acquisition time, 26 min. The product ion (TOF–MS-IDA-MS/MS) scanning method starting from the parent ion was adopted, and the second level was triggered using MMDF and DBS. The second level data acquisition was performed if the conditions were met.

In this experiment, QC samples were used to verify the method. Five QC samples were repeatedly injected before sequence injection to calibrate the instrument. The system was calibrated using QC samples during sequence injection after testing every five samples.

### Collection and processing of feces samples

Rat fecal samples were collected after 1 h from the last drug administration. The sample collection was as follows. Rat defecation was promoted using the tail-pick method. Rat feces were collected and placed into a 10-mL sterile tube; 2–3 pellets were collected, sealed, immediately frozen into liquid nitrogen, and stored at − 80 °C for subsequent use.

Ten fecal samples (from 10 different rats) from each group were randomly selected for *16S rRNA* gene sequencing analysis to determine the differences in the composition of gut microbiota between the control and OU model group. The E.Z.N.A.Stool DNA kit was used to extract the total bacterial DNA. The PCR amplification of the *16S rRNA* gene was performed using primers synthesized with barcodes based on the target sequences. Each experiment was performed in triplicate. The PCR amplicons of the same sample were mixed and detection was performed using 2% agarose gel electrophoresis. The PCR products from the gel were recovered using the AxyPrep DNA gel recovery kit and were eluted with Tris–HCL. The PCR products were detected and quantified using the QuantiFluor™-ST Blue Fluorescence Quantitative System (Promega, Madison, WI, USA) according to the preliminary quantitative results of electrophoresis and then mixed in the proportions corresponding to the sequencing volume requirements of each sample. Finally, the MiSeq library was constructed, and MiSeq sequencing was performed. PCR amplification of the hypervariable V3-V4 region of the bacterial *16S rRNA* gene was performed using the following primers: 338F 5′-ACTCCTACGGGAGGCAGCA-3′ and 806R 5′-GGACTACHVGGGTWTCTAAT-3′. The PCR reaction conditions were the following: initial denaturation at 95 °C for 3 min, followed by 27 cycles of denaturation at 95 °C for 30 s, annealing at 55 °C for 30 s, extension at 72 °C for 45 s, final extension at 72 °C for 10 min and 10 °C until taken out. The samples were sent to Shanghai Meiji Biomedical Technology Co., Ltd. (China) for sequencing.

### Correlation between metabolites and gut microbiota

Pharmacodynamics, metabolomics, and gut microbial analyses were used to assess the correlation between metabolites and gut microbiota in rat OU models. The data of gut metabolites and microbiota were combined to find the effects of B4 treatment on this correlation.

Statistical analysis was performed using SPSS (23.0) software (IBM company, Armonk, NY, USA). A two-level method was used to identify the key related substances. The datasets of gut microbial composition and plasma metabolites of the rats were extracted using Spearman correlation analysis. All the statistical analyses were two-sided, and a value of *P* < 0.05 was considered statistically significant. The corrected *P* value (false discovery rate = 0.05) was used to control the error of testing multiple hypotheses.

### Data processing and multivariate statistical analyses

Makeviewer software (Waters company, Milford, MA, USA) was used to import all the original liquid chromatography-mass spectrometry (LCMS) data for their processing and operations, such as peak alignment, peak identification, deconvolution, and data filtering. The processed data were then initially grouped and exported to SIMCA 14.1 (Umetrics Sweden company, Umea, Sweden) for principal component analysis (PCA) and orthogonal partial least square-discriminant analysis. The markers with variable important in projection (VIP) > 1 were screened according to the mathematical model orthogonal partial least square discriminant analysis (OPLS-DA). SPSS 23.0 was used to perform one-way analysis of variance (ANOVA). The differentially labeled compounds were selected using the established tags (VIP > 1 and *P* < 0.05). The potential biomarkers were selected and identified using online databases, such as Chemspider (http://www.chemspider.com/), Human Metabolomics Database (HMDB, http://www.hmdb.ca/), METLIN (http://metlin.scripps.edu), Mass Bank (https://massbank.eu/MassBank), and Kyoto Encyclopedia of Genes and Genomes (KEGG, http://www.kegg.jp). PeakView software (AB SCIEX company) was used to match the potential biomarkers with the secondary fragments to obtain the final identification results.

The online platform Pathway Analysis module of MetaboAnalyst 5.0 (http://www.metaboanalyst.ca) was used to perform the enrichment of the identified biomarkers to obtain the metabolic pathways related to OU disease. These results were combined with those of the KEGG database and a metabolic network was built. Finally, the metabolic markers and pathways in OU rat model and B4-treated rat model were determined.

High-dimensional biomarkers, including barcode based, metabolism, and classification, that distinguish between two or more biological conditions (or taxa) are identified by the Linear discriminant analysis Effect Size (LEfSe) (Sims et al. 2020). First, the significant difference in abundance among different groups was detected by the non-parametric factorial Kruskal–Wallis (KW) sum-rank test. Finally, the effect of the species abundance on the difference effect was estimated using LEfSe with linear discriminant analysis (LDA).

The paired-end reads were obtained using MiSeq sequencing (NCBI Sequence Read Archive database accession number: SRP437325). Next, they were spliced according to the overlapping sequences, and then, subjected to sequence quality control and filtration. The samples were distinguished and subjected to operational taxonomic unit (OTU) cluster and species taxonomy analyses. The multivariate statistical analysis partial least square discriminant analysis (PLS-DA) was used to identify the classification according to the observed or measured values of several variables. The specific analysis process was performed as follows: (1) the representative sequence of each operational taxonomic unit (OTU) was used for the classification, status, identification, and phylogenetic analysis. (2) The diversity of each sample was evaluated according to the abundance of OTUs in different samples. The curve reflected the sequencing depth meeting the standard. (3) The specific composition analysis of each sample and statistical difference among the groups were performed at different classification levels. (4) The multivariate statistical analysis of mathematical models was used to assess the differences in the structure of bacterial community among the different sample groups as well as the species associated to the differences. (5) An interaction network was built according to the distribution of species in each sample. (6) The *16S rRNA* gene sequencing was used to predict the metabolic function of gut microbial species in each sample. First, Phylogenetic Investigation of Communities by Reconstruction of Unobserved States (PICRUSt, https://cloud.majorbio.com/page/project/overview.html) was used to standardize the OTU abundance table. The PICRUSt process stores the Clusters of Orthologous Groups (COG) and KEGG Ortholog (KO) information corresponding to the Greengene ID. The standardization process was performed by the removal of the effect of copy number variations of the *16S rRNA* gene in the species genome. Then, the Greengene IDs were assigned to each OTU, and the COG family and KO information corresponding to OTU were obtained. The COG and KO abundances were calculated. The description of each COG and its function are obtained from the eggNOG database (http://eggnog.embl.de/) according to the COG database (http://www.ncbi.nlm.nih.gov/COG/). Moreover, the KEGG database (http://www.genome.jp/kegg/) was used to obtain the KO, pathway, and EC information.

## Results

### Rat’s OU area and H&E staining results

**Fig. 1:**
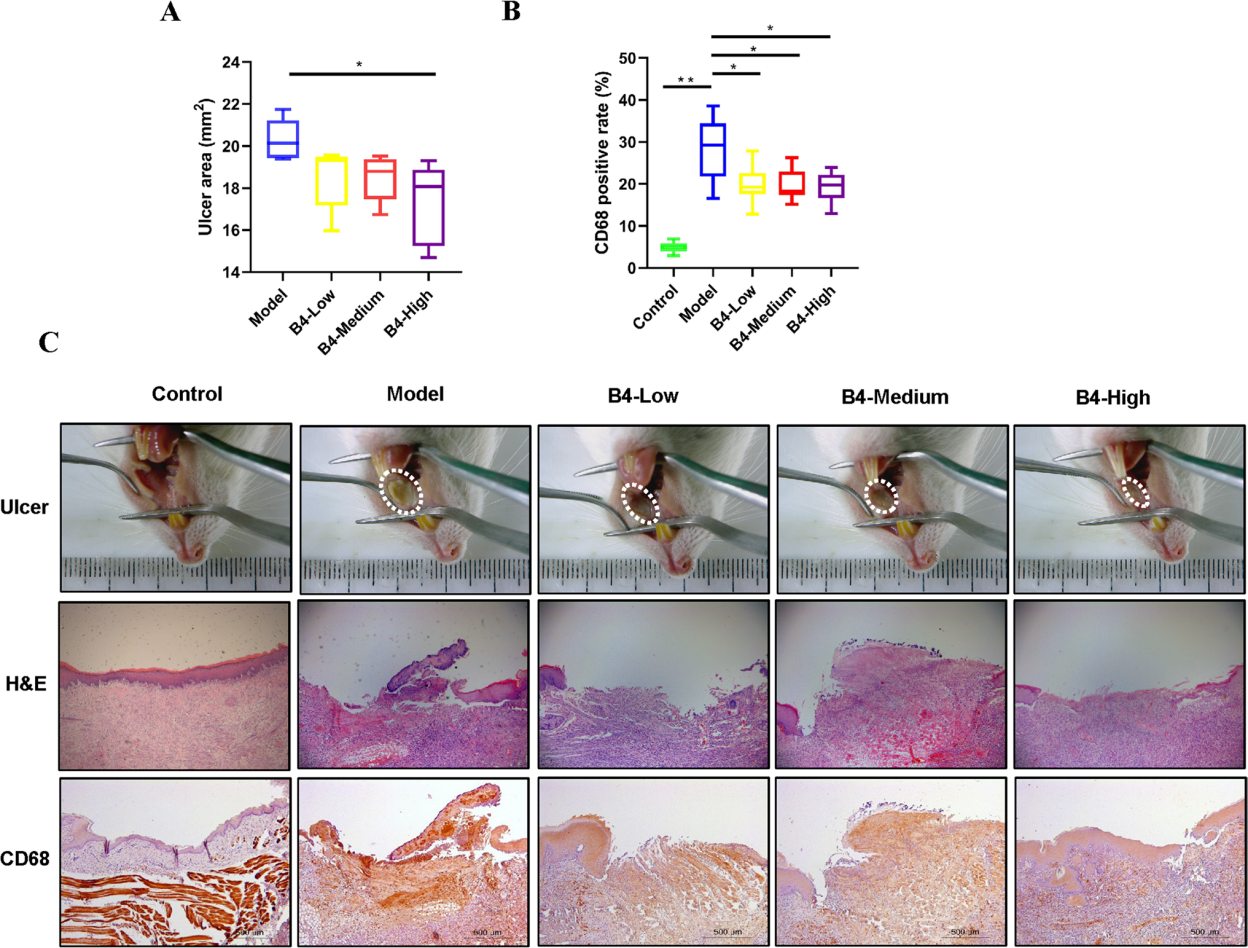
B4 alleviates inflammation and enhances ulcer healing in rats with oral ulcer. **A** Quantification of ulcer area. **B** Quantification of CD68 positive cells in the rat mucosal region of oral ulcer. **C** Representative images of macroscopic observation, histopathology, and CD68 staining of the oral mucosal region in OU rats on day 5 after B4 treatment. Macrographs of oral ulcer (upper), H&E staining of the oral ulcer (middle, X5), and immunohistochemical staining of CD68 in the oral ulcer (low, X5). Results are represented as mean ± SD (standard deviation), *n* = 8. *P < 0.05, **P < 0.01. Eight different biological replicates were analyzed per animal group

The OU area significantly increased in the model group compared to the control group (*P* < 0.01) (Fig. 1). The epithelium of the oral mucosa in the rats of the control group was intact with a clear structure, while the epithelium of the OU mucosa in the rats of the model group was damaged accompanied by irregular cellular arrangement, as revealed by the H&E results. Moreover, much inflammatory cell infiltration and red blood cell extravasation were present. The inflammatory cell infiltration in the oral mucosa of the treatment group rats was improved as compared to that in the model group, and new collagen fibrous tissues covered the injured mucosal area.

### IHC staining of rat oral mucosa after B4 suppository treatment

CD68 expression significantly increased in the oral mucosal tissues of the model group as compared to that in the control group (*P* < 0.01) and significantly decreased in the drug-administration groups (*P* < 0.05). CD68 expression was higher in the mucosal layer of the model group as compared to that in the control group, as shown in Fig. 1. More necrotic areas were also present in the model group. On the other hand, CD68 expression decreased in the mucosal layer of the rats of the treatment group.

### Screening and identification of potential OU biomarkers in rats

Clear differences in rat plasma samples of the control and OU model groups were observed, as shown by the multivariate statistical methods and PCA chart analysis, suggesting significant differences in the endogenous metabolites between the two groups. The VIP value in the OPLS-DA model reflected the contribution of different variable data to the metabolic differences between the two groups (Boccard and Rutledge 2013). The structures of 34 endogenous metabolites were identified by the analysis of the components of the previously mentioned potential biomarkers, and they mainly included bile acids, phospholipids, and sterols (Table 1). The heat map analysis of the relative content shown in Supplementary Fig. S2-1 further demonstrated the biomarkers related to OU disease and changes in the levels of each endogenous substance. The hot color in the heat map indicates relative content; the red and green colors the relatively low and high content, respectively. The darker red and green color intensity indicates the lower and higher relative contents, respectively. The rats were clearly divided into the control and OU model group according to the hierarchical cluster analysis (HCA) in the heat map.

**Table 1:** Identification of potential biomarkers of rat plasma samples between control group and OU model group

NO	Retention time	Name	Formula	Experimental mass	Ion mode	Mass error	MS/MS	HMDB	Levels
P1	2.64	3-Chloro-4-(dichloromethylene)-2,5-pyrrolidinedione	C_5_H_2_ClNO_2_	195.91137	[M + H-H2O]^+^	− 0.7	–	HMDB0034897	↓
P2	10.274	4-Hydroxybenzophenone	C_13_H_10_O_2_	205.08458	[M + Li]^+^	0.4	149.0212, 121.0363	HMDB0240708	↑
P3	9.71	2,5-Dichloro-4-oxohex-2-enedioate	C_6_H_4_Cl_2_O_5_	226.95086	[M + H]^+^	− 1.5	–	HMDB0060363	↓
P4	7.32	Dodecanoylcarnitine	C_19_H_37_NO_4_	344.27973	[M + H]^+^	− 0.2	298.2734, 226.2522, 73.0287	HMDB0002250	↓
P5	22.3	25-Azacholesterol	C_26_H_45_NO	352.33939	[M + H-2H2O]^+^	− 1.2	–	HMDB0001028	↑
P6	18.85	Lathosterol	C_27_H_46_O	369.35145	[M + H-H20]^+^	0.1	–	HMDB0001170	↑
P7	22.56	Clionasterol	C_29_H_50_O	397.38234	[M + H-H2O]^+^	− 0.9	–	HMDB0000649	↓
P8	14.51	7α-Hydroxy-3-oxo-5β-cholan-24-oic acid	C_24_H_38_O_4_	413.26637	[M + Na]^+^	− 0.8	–	HMDB0000503	↓
P9	20.17	NTP	C_6_H_15_O_13_P_3_	464.8913	[M + 2 K-H]^+^	− 6	313.2150, 295.1935, 226.9544, 158.9666	HMDB0060500	↑
P10	10.09	LysoPC(15:0/0:0)	C_23_H_48_NO_7_P	482.32362	[M + H]^+^	− 1.6	462.3117, 184.0727, 104.1070	HMDB0010381	↑
P11	11.43	DG(8:0/14:0/0:0)	C_25_H_48_O_5_	497.34338	[M + H + HCOONa]^+^	0.6	479.3336, 184.0738, 104.1071	HMDB0092913	↑
P12	15.46	LysoPC(18:0/0:0)	C_26_H_54_NO_7_P	546.35216	[M + Na]^+^	− 2.1	487.2789, 341.3049, 146.9814	HMDB0010384	↑
P13	11.42	LysoPC(18:S(6Z,9Z,12Z)/0:0)	C_26_H_48_NO_7_P	586.30852	[M + H + HCOONa]^+^	− 2.4	518.3215, 459.2479, 437.2021, 104.1066	HMDB0010387	↑
P14	15.46	7,12-Dihydroxy-3,11,15,23-tetraoxolanost-8-en-26-oic acid	C_30_H_42_O_8_	615.3425	[M + IsoProp + Na + H]^+^	− 0.3	547.3548, 488.2835, 146.9812, 104.1075	HMDB0033023	↑
P15	15.47	(3a,5b)-24-Oxo-24-[(2-sulfoethyl)amino]cholan-3-yl-β-D-glucopyranosiduronic acid	C_32_H_53_NO_11_S	682.3261	[M + Na]^+^	0.1	546.3522, 487.2781, 104.1069	HMDB0002429	↑
P16	20.35	PE-NME(16:1(9Z)/22:2(13Z,16Z))	C_44_H_82_NO_8_P	806.56848	[M + Na]^+^	− 1.3	679.4180, 622.4826, 184.0734	HMDB0113077	↓
P17	22.11	PG(18:0/20:3(5Z,8Z,11Z))	C_44_H_81_O_10_P	807.57102	[M + Li]^+^	− 0.7	–	HMDB0010608	↓
P18	21.43	PC(14:0/22:2(13Z,16Z))	C_44_H_84_NO_8_P	830.56127	[M + 2Na-H]^+^	− 7.5	–	HMDB0007888	↓
P19	20.33	PE(DiMe(11,3)/DiMe(11,5))	C_47_H_82_NO_10_P	896.53778	[M + 2Na-H]^+^	− 0.8	–	HMDB0061472	↓
P20	1.26	1,2-Dichloroethane	C_2_H_4_Cl_2_	96.96199	[M-H]^−^	− 0.1	79.9598, 63.9629	HMDB0029571	↓
P21	8.67	*N*-Carboxyethyl-γ-aminobutyric acid	C_7_H_13_NO_4_	194.0823	[M + F]^−^	− 2.1	179.0579, 164.0830, 108.0222	HMDB0002201	↑
P22	5.2	2-Hydroxycinnamic acid	C_9_H_8_O_3_	209.04547	[M + FA-H]^−^	0.4	165.0556, 141.0937, 121.0292	HMDB0002641	↑
P23	6.87	3-Ethenyl-4-hydroxy-2,5-dimethylhex-5-en-2-yl acetate	C_12_H_20_O_3_	211.13408	[M-H]^−^	− 0.1	167.1427, 151.1143, 111.0803	HMDB0041572	↑
P24	8.17	Valdiate	C_17_H_26_O_5_	309.17089	[M-H]^−^	0.3	281.1754, 219.1749, 162.1042, 59.0185	HMDB0040980	↑
P25	8.94	Stearoylglycine	C_20_H_39_NO_3_	378.24174	[M + K-2H]^−^	0.9	298.1153, 242.9456, 78.9614	HMDB0013308	↓
P26	6.57	15,17-Dihydroxy-11-methoxy-6,8,20-trioxapentacyclo-icosa-1(12),2(9),10,14,16,18-hexaen-13-one	C_18_H_14_O_7_	401.08807	[M + HAC-H]^−^	− 0.1	357.0620, 313.0717, 269.0814, 121.0299	HMDB0128881	↑
P27	16.62	Stearoylcarnitine	C_25_H_49_NO_4_	464.31607	[M + K-2H]^−^	0.4	421.2762, 267.2685, 196.0385, 78.9643	HMDB0000848	↓
P28	6.57	Apiosylglucosyl 4-hydroxybenzoate	C_18_H_24_O_12_	469.07586	[M + K-2H]^−^	− 0.3	401.0890, 313.0717, 121.0303	HMDB0035318	↓
P29	8.4	7-Chloro-3,3′,4′,5,6,8-hexamethoxyflavone	C_21_H_21_ClO_8_	471.06281	[M + Cl]^−^	− 2.1	335.2207, 265.9551, 129.9744	HMDB0032658	↓
P30	7.42	Deoxynivalenol 3-glucoside	C_12_H_12_N_2_O_2_	477.17775	[M + F]^−^	− 0.7	409.2897, 340.8955, 272.8877	HMDB0039852	↑
P31	8.56	1-Nitro-7-glutathionyl-8-hydroxy-7,8-dihydronaphthalene	C_20_H_24_N_4_O_9_S	517.1009	[M + Na-2H]^−^	− 1	401.0886, 313.0707, 121.0297	HMDB0060329	↓
P32	10.19	LysoPE(0:0/22:6(4Z,7Z,10Z,13Z,16Z,19Z))	C_27_H_44_NO_7_P	524.27964	[M-H]^−^	− 0.9	464.3071, 327.2309, 196.0369	HMDB0011496	↑
P33	10.03	*N*-Arachidonoyl tyrosine	C_29_H_41_NO_4_	526.31672	[M + HAC-H]^−^	− 0.7	377.2085, 241.2173	HMDB0062331	↑
P34	13.06	LysoPC(P-18:0/0:0)	C_26_H_54_NO_6_P	552.36871	[M + FA-H]^−^	− 0.7	492.3494, 146.9599	HMDB0013122	↑

*NTP*, nucleoside triphosphate; “↑”—compared with the control group, the relative content of compounds in the OU model group was upregulated; “↓”—compared with the control group, the relative content of compounds in the OU model group was downregulated

Per animal group, 8 different biological replicates were analyzed

**Fig. 2:**
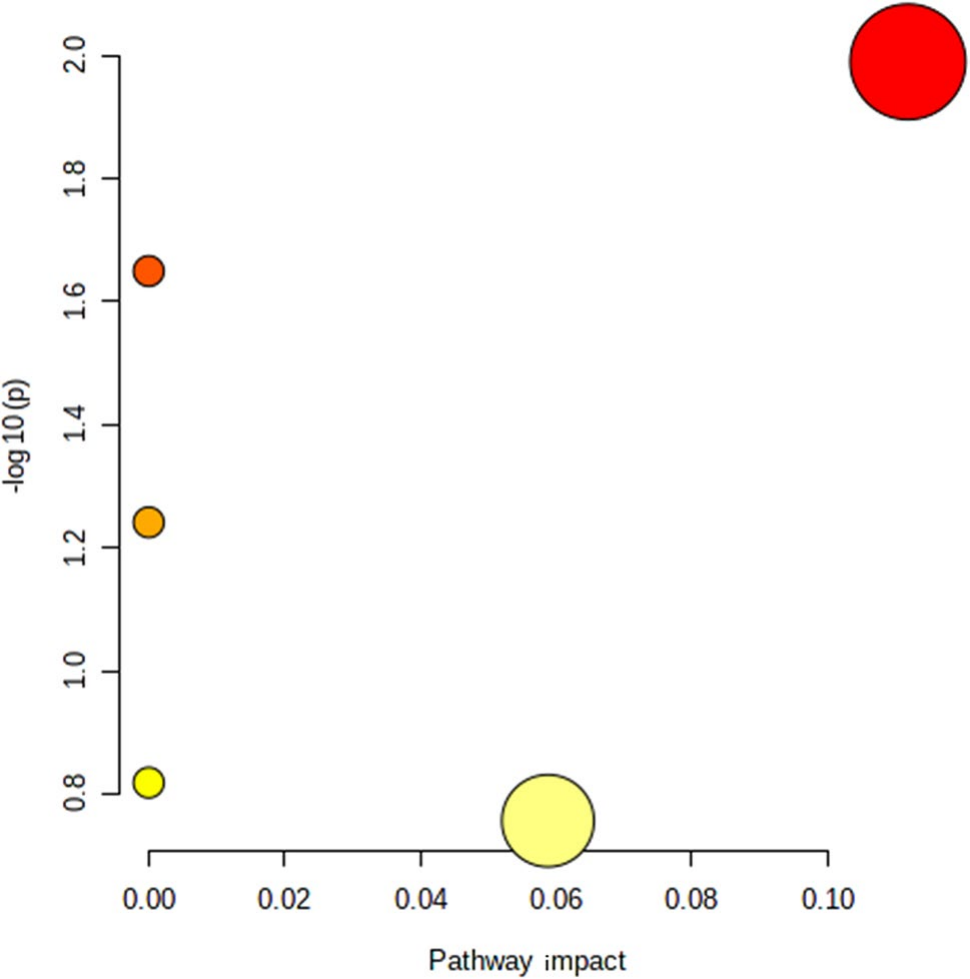
Serum metabonomics study of rat oral ulcer model. Enrichment map of metabolic pathways based on serum biomarkers of oral ulcer disease model. The size of the bubble represents the impact of the pathway, and the color represents different pathways. Eight different biological replicates were analyzed per animal group

The enrichment topological analysis of the previously identified 34 potential biomarkers was performed using the MetaboAnalyst online platform and KEGG database, as shown in Fig. 2. The potential biomarkers of the OU model were mainly related to the metabolisms of glycerophospholipid, linoleic acid, α-linolenic acid, arachidonic acid, and steroid biosynthesis.

## Analysis of metabolic profiles after the administration of *Pulsatilla* saponin B4

The data of five groups, such as control, OU model, B4 low-dose, B4 medium-dose, and B4 high-dose groups, were imported to SIMCA-P 14.1 to obtain a comprehensive and holistic metabolic profile and study the effect of different doses of *Pulsatilla* saponin B4 on the endogenous substances in the plasma of the OU rat model (in Supplementary Fig. S2-2). A significant difference in the metabolic profile of the plasma samples of the control and OU model group was observed in the positive and negative ion mode by PCA analysis. The results of the B4 low-dose group were between those of the OU model and control group, indicating that the administration of B4 induced a tendency of the overall metabolic profile of the rats towards that of the control group. The five groups of samples were clustered together in a supervised mode, as shown by the OPLS-DA analysis. The results of the B4 low-dose group were between the OU model and control group, indicating that B4 low-dose had a therapeutic effect on OU, which was further confirmed in the drug efficacy experiments.

## Regulatory effects of *Pulsatilla* saponin B4 on potential biomarkers

The OU-related potential biomarkers were identified by the early multivariate statistical model analysis. B4 treatment reduced the levels of biomarkers in the serum of OU model rats. The changes in the metabolic levels of biomarkers, such as the levels of 21 of 34 potential biomarkers tended to be normal after B4 treatment, as revealed by the box plot drawn that clarify the pathogenesis of the OU disease model and the effects of B4 on OU shown in Supplementary Fig. S2-3. The levels of stearoylcarnitine (stearoyl carnitine), lathosterol (sitosterol), 3-chloro-4-(dichloromethylene)-2,5-pyrrolidinedione (3-chloro-4-(dichloromethylene)-2,5-pyrrolidinedione), stearoylglycine (stearoylglycine), lysophosphatidylcholine (15:/0:0), *N*-arachidonoyl tyrosine (*N*-arachidonic acid tyrosine), and 4-hydroxybenzophenone (4-hydroxybenzophenone ketone) in the serum significantly decreased and were close to those in the control group. These biomarkers were involved in biological metabolic pathways such as lipids, arachidonic acid, and glycerophospholipids as revealed by the MetaboAnalyst analysis website.

### Composition analysis of gut microbiota

**Fig. 3:**
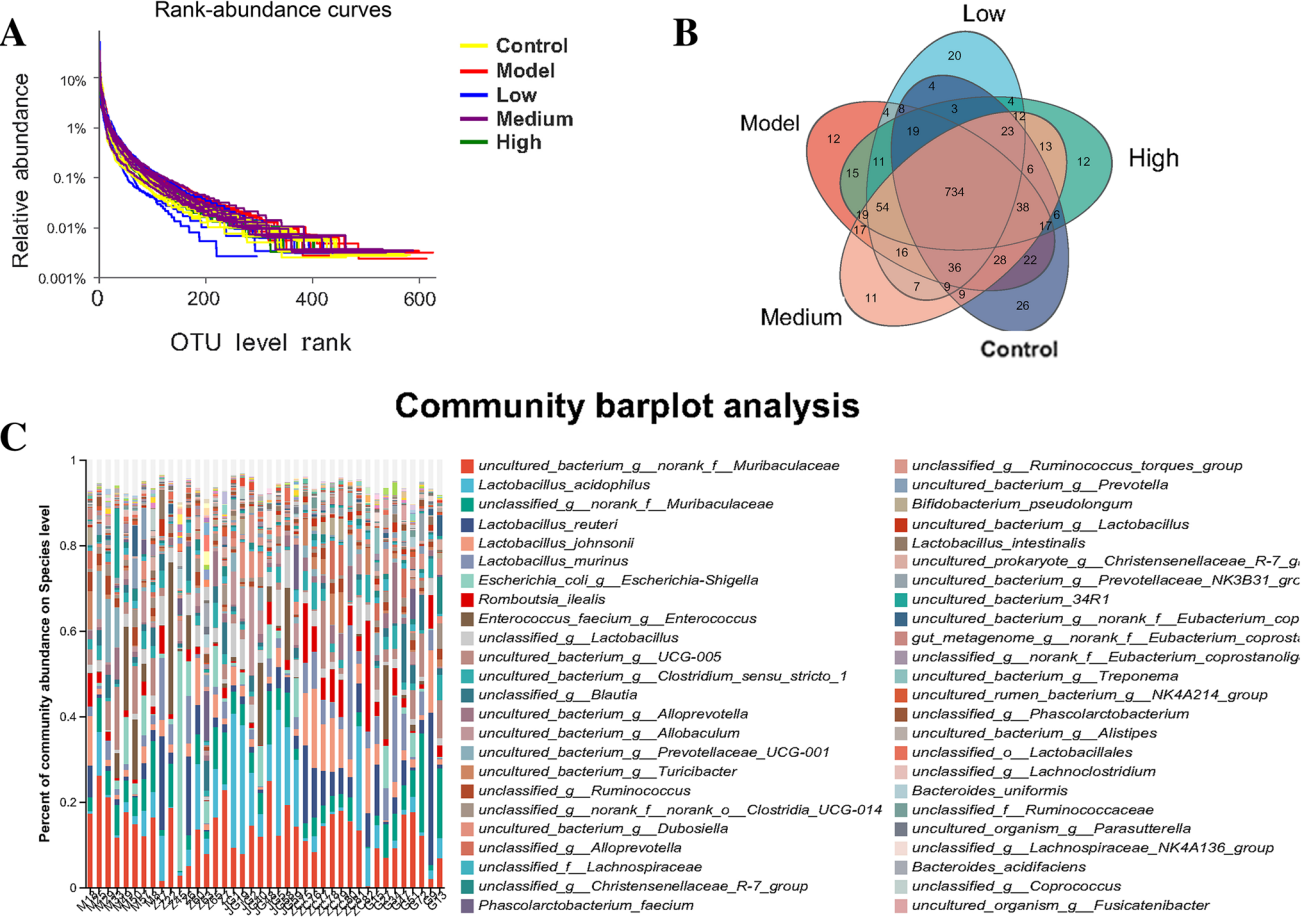
Evaluation of rat intestinal bacteriome in the overall sample. **A** Dilution curve of intestinal microbial samples of each group. **B** Venn diagram of intestinal microbial samples of each group. **C** Abundance of the overall species of the population of intestinal bacteria at the level of phylum, family, class, order, and genus. Eight different biological replicates were analyzed per animal group

The diversity of gut microbiota was analyzed by plotting a rank-abundance curve, which involves the count of the number of sequences contained in each OTU in each sample and the subsequent sort of the OTUs in descending order of abundance. This curve was plotted using the rank of OTU as abscissa and the number of sequences contained in each OTU as ordinate, and explains the two aspects of diversity, such as species richness and community evenness. The species richness is represented by the curve width in the horizontal direction (x-axis); the larger the curve on the x-axis, the higher the species richness. The smoothness of the curve represents the uniformity of the communities in a sample; the smoother the curve, the more uniform the species distribution (Lzask and Pavoine 2012). The blue and yellow curves, which represented the low-dose and control groups, respectively, rapidly and abruptly dropped, suggesting that the sample had a high proportion of dominant bacteria and low diversity, as shown in Fig. 3. Moreover, the composition of gut microbiota in the low-dose group was closer to that in the control group.

**Fig. 4:**
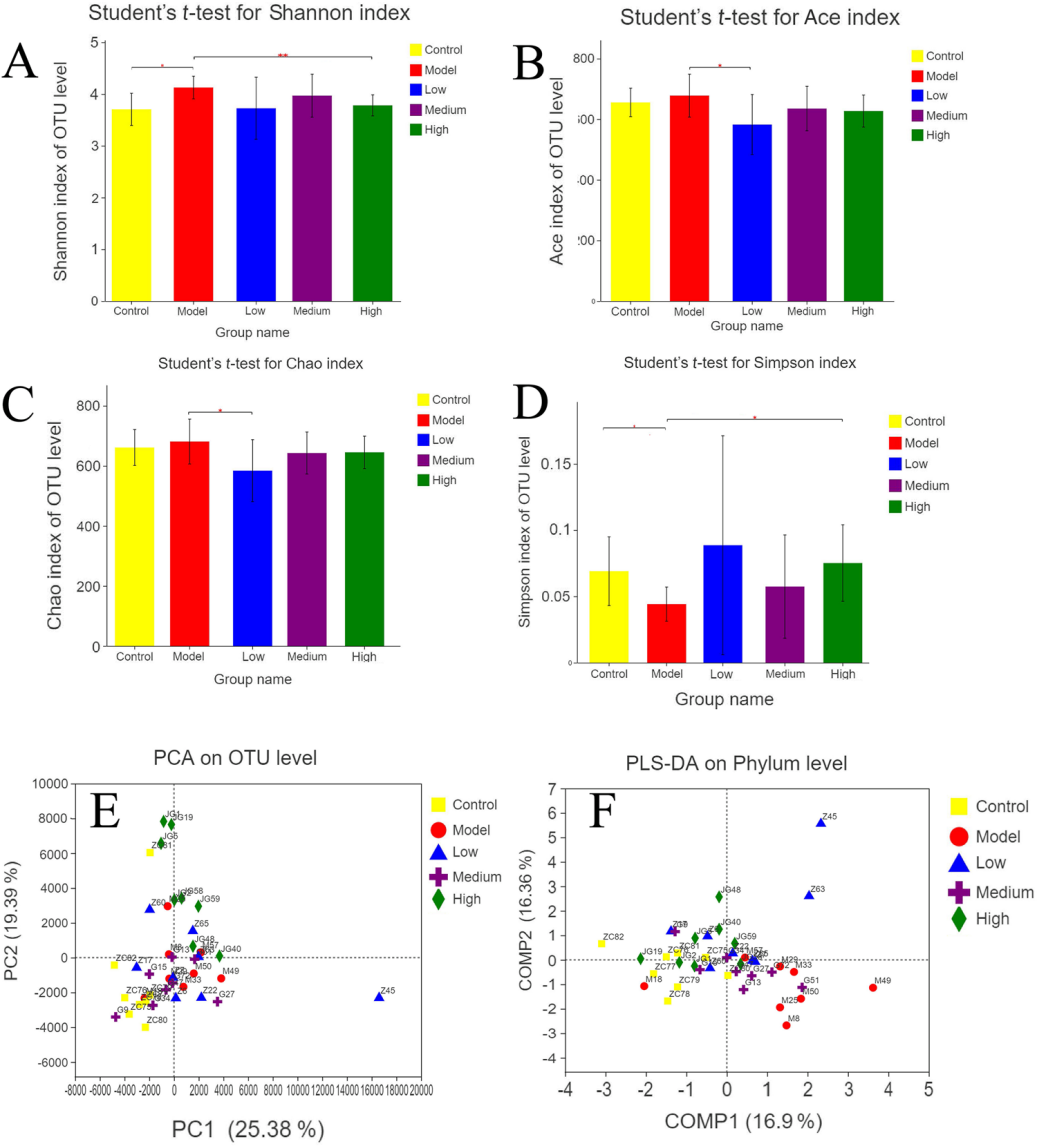
Microbiome data analysis and function classification. **A**–**D** Rat microbiome ɑ diversity analysis (Shannon, Ace, Chao, Simpson); **E**, **F** rat microbiome β diversity analysis (PCA, OPLS-DA at the gate level); eight different biological replicates were analyzed per animal group

The abundance of gut microbiota in the OU model group was higher than that in the control group, while the abundance of species in the B4 groups showed an increasing tendency, approaching the abundance of species in the control group, as shown in Fig. 3 and Supplementary Figs. S3-1 and S3-2. Moreover, the species abundance in the low-dose and high-dose B4 groups was closer to that in the control group. Among the 1215 OTUs clustered with 97% similarity, 734 OTUs were shared by the control group, OU model group, and B4 treatment groups (Fig. 4, Supplementary Fig. S4); 22 OTUs were shared by the control and OU model group; 36 OTUs were shared by the control and treatment group; and 54 OUTs were shared by the OU model and treatment group. These results indicate a significant functional difference in gut microbiota among the control, treatment, and OU model group.

**Fig. 5:**
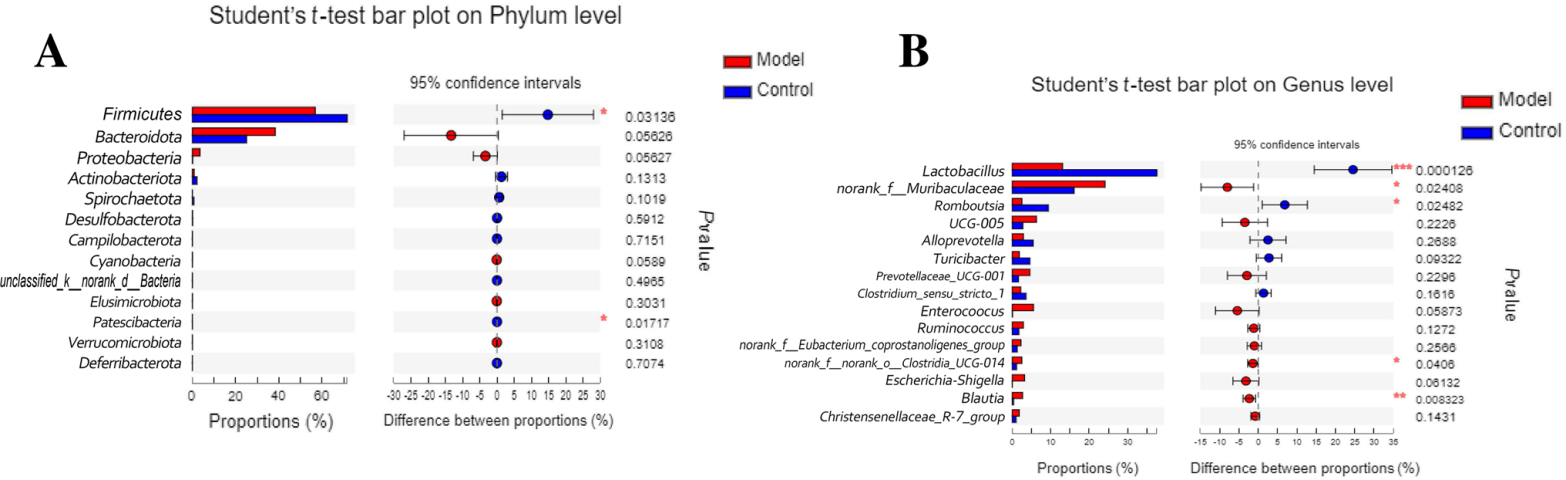
Analysis of the correlation between biomarkers and intestinal microbiome. **A** Analysis of the difference of fecal microbiome between the control group and model group at the phylum level. **B** Analysis of the difference between the fecal microbiome of the control group and model group at the genus level. Eight different biological replicates were analyzed per animal group

The composition and species abundance of gut microbiota were analyzed in each sample at the level of phylum, class, order, family, and genus (Fig. 5). The bar graphs and heat maps showed the changes in the gut microbiota of each group at the phylum level, revealing significant differences in the composition of gut microbiota between the control and OU model group. Moreover, the composition of gut microbiota in the OU model rats after the treatment with B4 was similar to that in the control group, suggesting that B4 reversed the composition of gut microbiota to a certain extent.

### Alpha diversity analysis of gut microbiota

The abundance and diversity of gut microbiota were analyzed using single-sample diversity (alpha diversity) analysis, which included a series of statistical indices. The number of species in a community without considering the richness or abundance of each species in the community is indicated by the Chao and Ace indices. The diversity of a community, which is affected by species richness and evenness is indicated by the Shannon and Simpson indices. When the richness involves the same species, the greater the species uniformity in a community, the greater the diversity of the community (Cheng et al. 2021a, b).

No significant difference in OTU abundance (Chao and Ace index) in the three groups was found after the analysis of the diversity indices of gut microbiota in the OU model, control, and B4 groups. However, the community diversity indices were significantly different between the control and OU model group [Simpson index (*P* = 0.02943, 0.069057 ± 0.025965 vs 0.04419 ± 0.012929) and Shannon index (*P* = 0.007214, 3.7048 ± 0.31016 vs 4.1267 ± 0.21938)]. This result suggested that the diversity of gut microbiota in the model group was higher than that in the control group. Moreover, the diversity of gut microbiota in the administration group was similar to that of the control group, while the diversity of gut microbiota in the OU rat model treated with high-dose B4 was significantly different from that of the model group.

### Beta diversity analysis of gut microbiota

The structure of gut microbiota in the control, administration, and OU model group was significantly different, as revealed by the beta diversity analysis of gut microbiota. The OTU information of each sample was constructed using the UniFrac distance matrix to further analyze the difference in the composition of gut microbiota among the five groups. Based on this matrix, the principal coordinates analysis (PCoA) was performed, revealing the difference and distance among the samples by analyzing the composition of OTU in different samples. The results showed the successful establishment of the OU rat model since the samples in the control and OU model group were in different regions with clear boundaries. The samples of the three treatment groups were scattered between the control and OU model group. This indicated that the administration of B4 modified the gut microbiota of the OU rat model. Moreover, some samples moved closer to the control group to a certain extent.

PLS-DA is a supervised analysis method requiring the grouping of test samples based on categories. The calculations of the mathematical model allow the distinction among groups, ignore the random differences within the groups, and highlight the system differences among groups. The observation factor is much larger than the number of samples and it has a good performance in distinguishing samples (Chen et al. 2020a, b). The PLS-DA diagram distinguished the samples in each group according to the distance. The samples of the model and control group had indeed a certain distance difference, while those of the administration group were scattered between the model and control group. These results suggested that B4 treatment exerted a therapeutic effect on the composition of gut microbiota in OU rats.

### Species difference analysis

The analysis of the species difference among the groups was performed according to the obtained abundance of gut microbiota. Strict statistical methods were used to perform the hypothesis tests on the species difference among the gut microbiota of different groups. The significant level of difference among the species abundances was evaluated.

LDA was performed according to the taxonomic composition and grouping conditions, and the most dominant communities or species with significant differences in the sample were identified by the size of the effects. The results presented in a cladogram showed a significant difference among all groups (Supplementary Figs. S5 and S6). The low-dose group significantly affected the bacterial abundance of 43 genus levels (*P* < 0.05 vs OU model), and the medium-dose group significantly affected the bacterial abundance of 29 genus levels (*P* < 0.05 vs OU model), while the high-dose group significantly affected the abundance of 22 genera (*P* < 0.05 vs OU model).

### Prediction and analysis of gut microbial function using PICRUSt

The abundance was calculated based on the abundance of each functional category. In addition, the three-level information on metabolic pathways and the abundance table for each level were obtained using PICRUSt. The COG function classification mainly contained carbohydrate transport and metabolism, amino acid transport and metabolism, replication, recombination and repair, translation, ribosomal structure and biogenesis, and transcription. The results showed that the OU rat model state changed, including amino acid metabolism, energy metabolism, lipid transport, nucleotide metabolism and transport, and signal transduction.

### Analysis of the correlation between metabolite and gut microbiota

The abundance dataset was subjected to Spearman correlation analysis to estimate the correlation between the differential metabolites and gut microbiota at the phylum, genus, and species levels. The significant correlation between metabolites and gut microbiota was evaluated by the comprehensive analysis of the *R* value and *P* value. The variable abundance and biological significance as key correlation pair were identified by the Spearman correlation analysis, which was performed on the abundance dataset to estimate the correlation between the differential metabolites and gut microbiota at the phylum level. Pearson correlation analysis showed that “lysopc(15:0/0:0)-*Bacteroidota*,” “stearoylglycine-*Clostridia*,” “*N*-arachidonoyl tyrosine-*Lactobacillus*,” “3-chloro-4-(dichloromethylene)-2,5-pyrrolidinedione-*Romboutsia*,” “3-chloro-4-(dichloromethylene)-2,5-pyrrolidinedione-*Alloprevotella*,” “lysopc(15:0/0:0)-*Lactobacillus*,” and “lysopc(15:0/0:0)-*Ruminococcus*” were the key metabolite and gut microbiota pairs from the perspective of the variable abundance and biological significance, which were associated with inflammation, immune mechanisms, and wound healing. *Lactobacillus* and *Romboutsia* were positively correlated with the identified compounds produced by lipid and fatty acid metabolism, while *Enterococcus* and *Escherichia-Shigella* were negatively correlated with these compounds.

## Discussion

The progression of several diseases is related to the changes in metabolomics and gut microbiota composition. The Chinese herbal medicine Vladimiriae Radix exerts therapeutic effects on ulcerative colitis by the regulation of metabolic abnormalities and the improvement in the accumulation of metabolites in the gut microbiota (Yu et al. 2021). The combination of the extracts of *Rehmannia glutinosa* and *Cornus officinalis* significantly improves kidney function indices and restores the composition of gut microbiota and endogenous metabolites (Zhang et al. 2021). Li et al. (2021) explored the mechanism of immunoglobulin A nephropathy by a combination of gut microbiota analysis and metabonomics and rats were treated with the blended traditional Chinese herbal medicine Zhen-Wu-Tang (which consists of five ingredients: *Poria cocos*, peony, *Atractylodes macrocephala*, ginger, and aconite), which resulted in a significant improvement in the disease condition. The abundance of gut microbiota changes after suppository administration, and the produced gut metabolites enter the bloodstream, thereby altering the metabolic profile (Crouwel et al. 2021; Ebenebe et al. 2021). However, the metabonomics of OU and changes in gut microbiota associated with it are not yet clear. In this study, we established a rat model of oral ulcer to explore the metabonomics and intestinal microbial changes of rats after B4 suppository administration, so as to explore the mechanism of action of B4 suppository in the treatment of oral ulcer rats.

Lipid and fatty acid metabolisms produce metabolites that are closely related to immune and inflammatory reactions. *Lactobacillus* present in the human body produces many beneficial substances and regulates the human immune system (Liang 2021). *Romboutsia* has the same symbiotic association with the human intestine (Cheng et al. 2021a, b). The gut of healthy individuals contains a high abundance of *Romboutsia* (Cheng et al. 2021a, b). The pathogenicity of *Enterococcus* has been reported by several studies in recent years (Ferchichi et al. 2021; Caballero-Granado et al. 2001). Indeed, *Enterococcus faecalis* produces the polymorphonuclear leukocyte chemotactic factor that mediates, at least partially, the inflammatory response associated with enterococcal infection (Caballero-Granado et al. 2001). In the present study, *Escherichia-Shigella* showed opposite richness trends in the model group compared to probiotics such as *Lactobacillus*, suggesting that *Escherichia-Shigella* mediated the associated inflammatory response.

Pei and He (2021) reported that anemoside B4 improves sepsis by alleviating the inflammatory reaction, apoptosis, and oxidative stress. Ma et al. (2020) showed that the therapeutic effect of B4 for ulcerative colitis involved inflammatory pathways; indeed, B4 improves macrophage activity in rats induced by lipopolysaccharide (LPS). Kang et al. (2019) reported that B4 alleviates LPS-induced kidney and lung inflammation and improves immunity in rats by inhibiting the nuclear factor kappa-light-chain-enhancer of activated B cells (NF-κB) pathway. These results might suggest that the potential mechanism of B4 to improve OU might be the involvement of the immune and inflammatory system. The administration of B4 in this study improved the pathogenic gut microbial environment in OU disease. B4 also decreased the expression of CD68 in the blood, indicating that OU was closely related to macrophage activity.

This study is the first investigating the correlation between OU and gut microbiota and introduced metabonomics from the perspective of endogenous metabolites. A close correlation between metabolites and gut microbiota in OU disease was successfully identified, as well as the effects of B4 on the metabolites and gut microbiota. The results showed that B4, an excellent anti-inflammatory monomer, exerted a therapeutic effect through the microorganism blood immune system, thus providing insights into the understanding of the mechanism of OU disease, posing a base for the development of *Pulsatilla* saponin B4 drug.

## Supplementary Information

Below is the link to the electronic supplementary material.

Supplementary file1 (PDF 7684 KB)

## Data Availability

The data (the accession number SRP437325) that support the findings of this study are available in the supplementary material of this article. The data used and/or investigated during the present study are available from the corresponding author upon reasonable request.
