# Supplementary material for: Pulchinenoside B4 ameliorates oral ulcers in rats by modulating gut microbiota and metabolites
Source: Appl Microbiol Biotechnol. 2024 Apr 9;108:292. doi: 10.1007/s00253-024-13099-1 (PMC11003895; doi:10.1007/s00253-024-13099-1)
Supplement: Supplementary file 1 [file 253_2024_13099_MOESM1_ESM.pdf]

Supplemental Material

Journal name: *Applied Microbiology and Biotechnology*

Manuscript Title: Pulchinenoside B4 ameliorates oral ulcers in rats by modulating gut microbiota and metabolites

The names of the authors: Dewei Luo, Li Yan, Zhujun Wang, Xiaofan Ji, Na Pei, Jing Jia, Yingying Luo, Hui Ouyang, Shiling Yang, Yuling Feng

The affiliations and addresses of the authors:

1.Jiangxi University of Traditional Chinese Medicine, No.818 Yunwan Road, Nanchang 330002, PR China

2.State Key Laboratory of Innovative Drug and Efficient Energy-Saving Pharmaceutical Equipment, No. 56 Yangming Road, Nanchang 330006, PR China

3.Research Center of Natural Resources of Chinese Medicinal Materials and Ethnic Medicine, Jiangxi University of Traditional Chinese Medicine, No.818 Yunwan Road. Nanchang 330004, PR China

4.Xinyu University, No.2666 Yangguang Road, Xinyu 338004, PR China

Corresponding authors: huiouyang@163.com, fengyulin2003@126.com, luoying0302@163.com.

H&E staining protocol

OU tissue sections were deparaffinized with xylene and washed with various levels of ethanol in the following steps: Sections were stained with hematoxylin solution for 3 to 5min, then differentiated with

differentiation solution, stained with back blue solution (0.4% ammonia), and finally rinsed with tap water. The rinsed sections were treated in absolute ethanol for 5 min(5 min each time, repeated 3 times) and xylene for 5 min(5 min each time, repeated 2 times) to make them transparent, and then the sections were dried and sealed with neutral glue and photographed under an electron microscope for histopathological changes.

Figures:

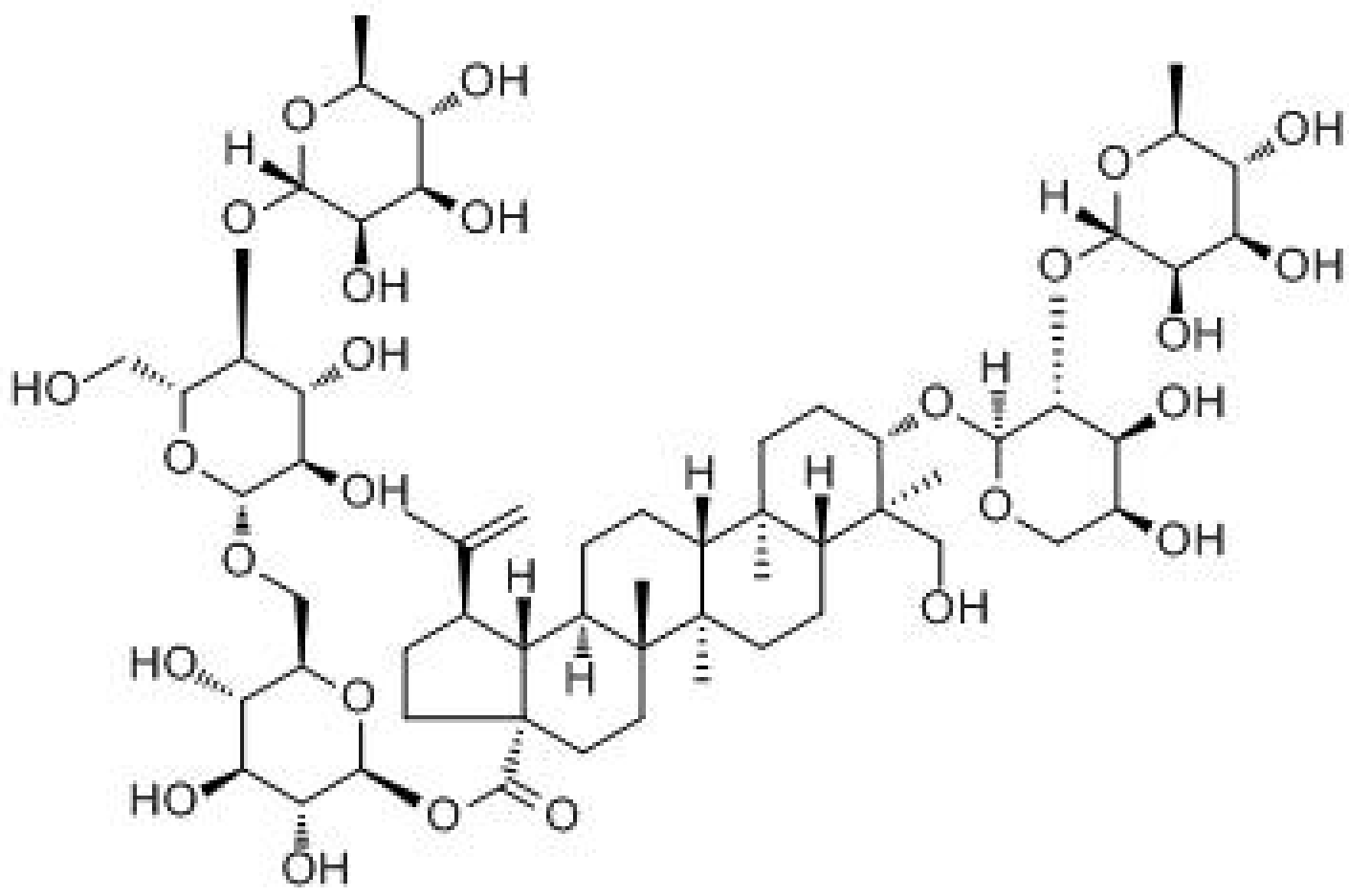

Fig. S1. *Pulsatilla* saponin B4 structure. Structure reference vital genesis network (https://www.chemsrc.com/cas/129741-57-7_895739.html)

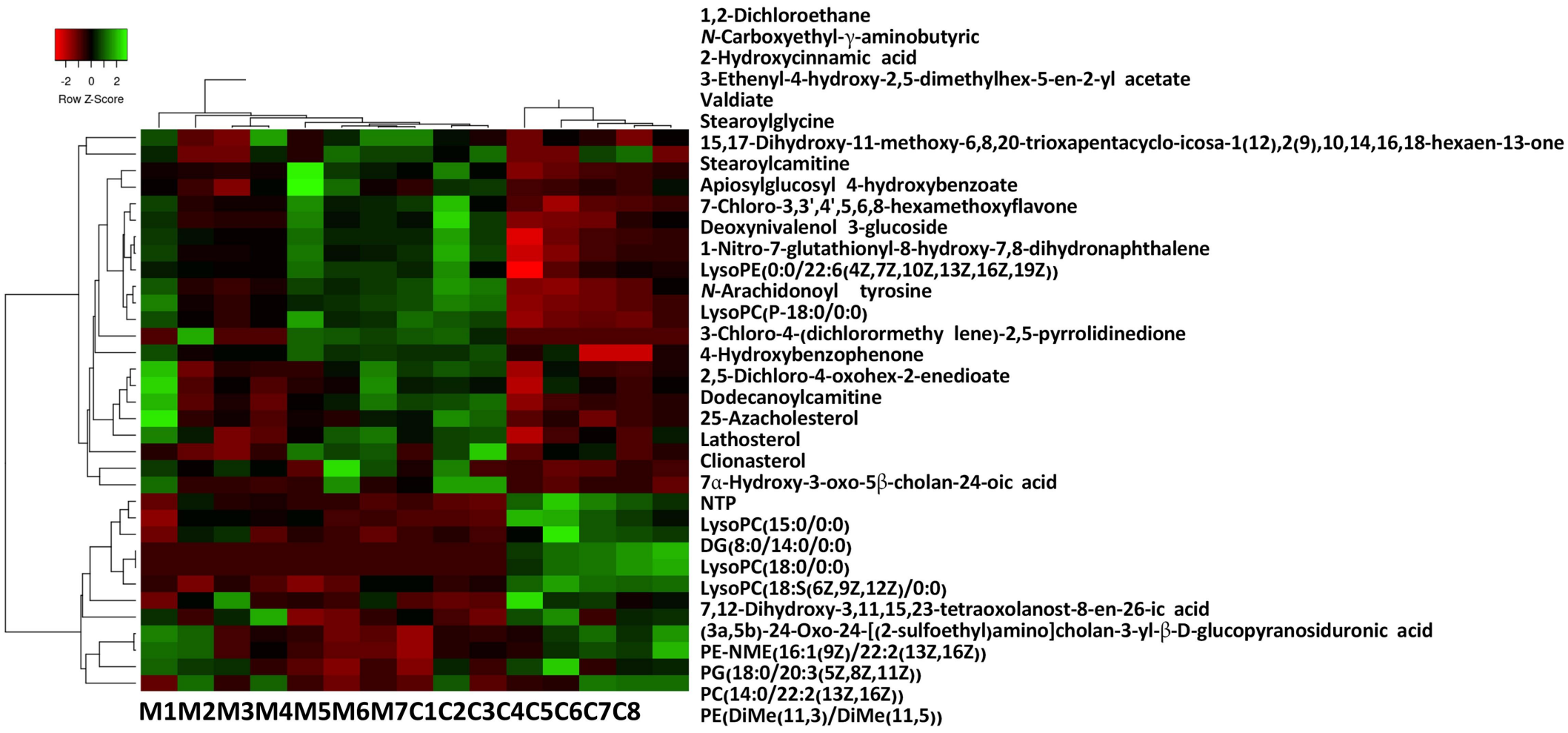

Fig. S2-1. Serum metabonomics study of rat oral ulcer model with clustering fever related to rats in normal and OU model groups.

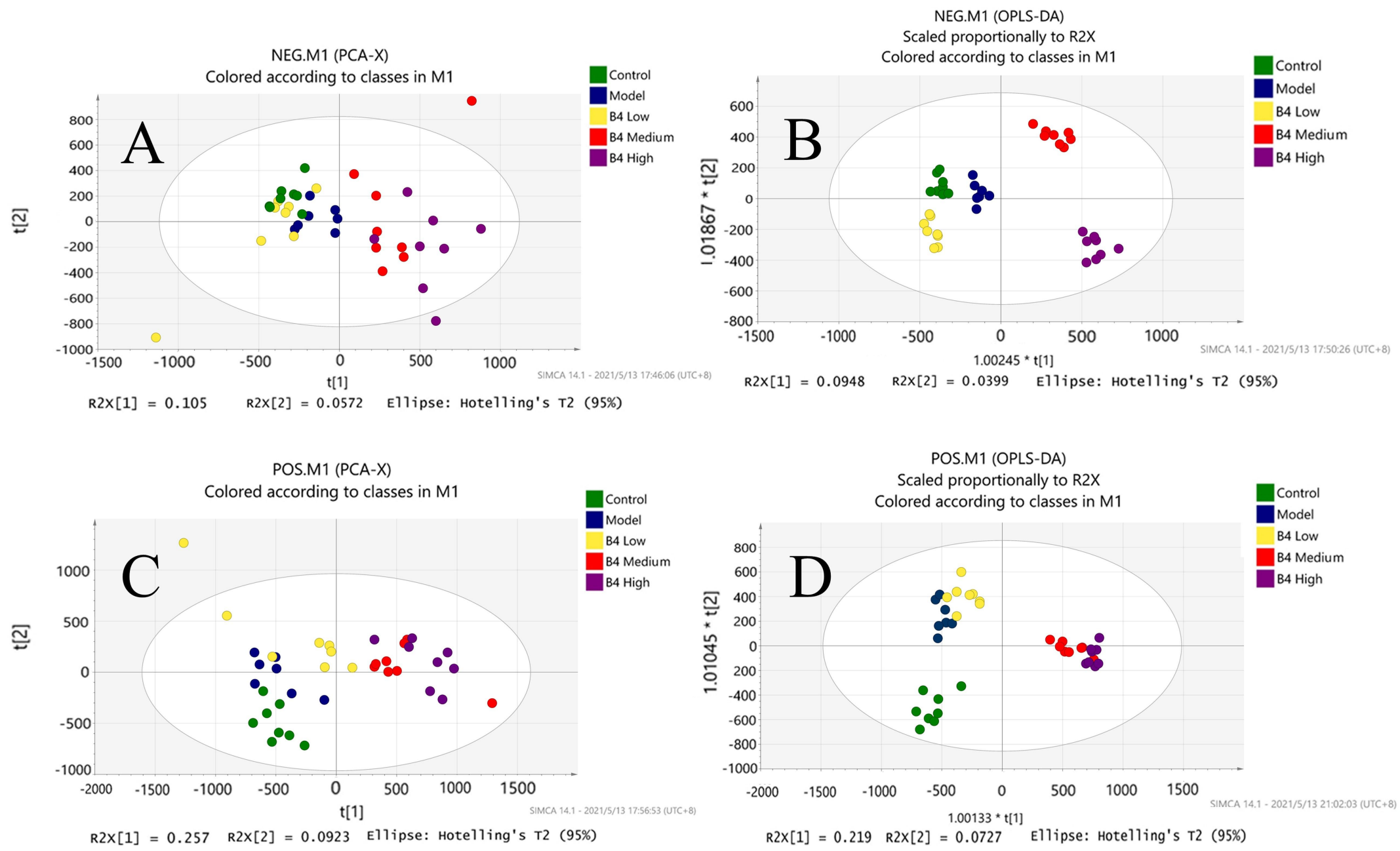

Fig. S2-2. Serum metabonomics study of rat oral ulcer model. A: PCA profile of each group of serum samples in negative ion mode. B: OPLS-DA profile of each group of serum samples in negative ion mode. C: PCA of each group of serum samples in positive ion mode contour map. D: OPLS-DA contour map of each group of serum samples in positive ion mode.

G

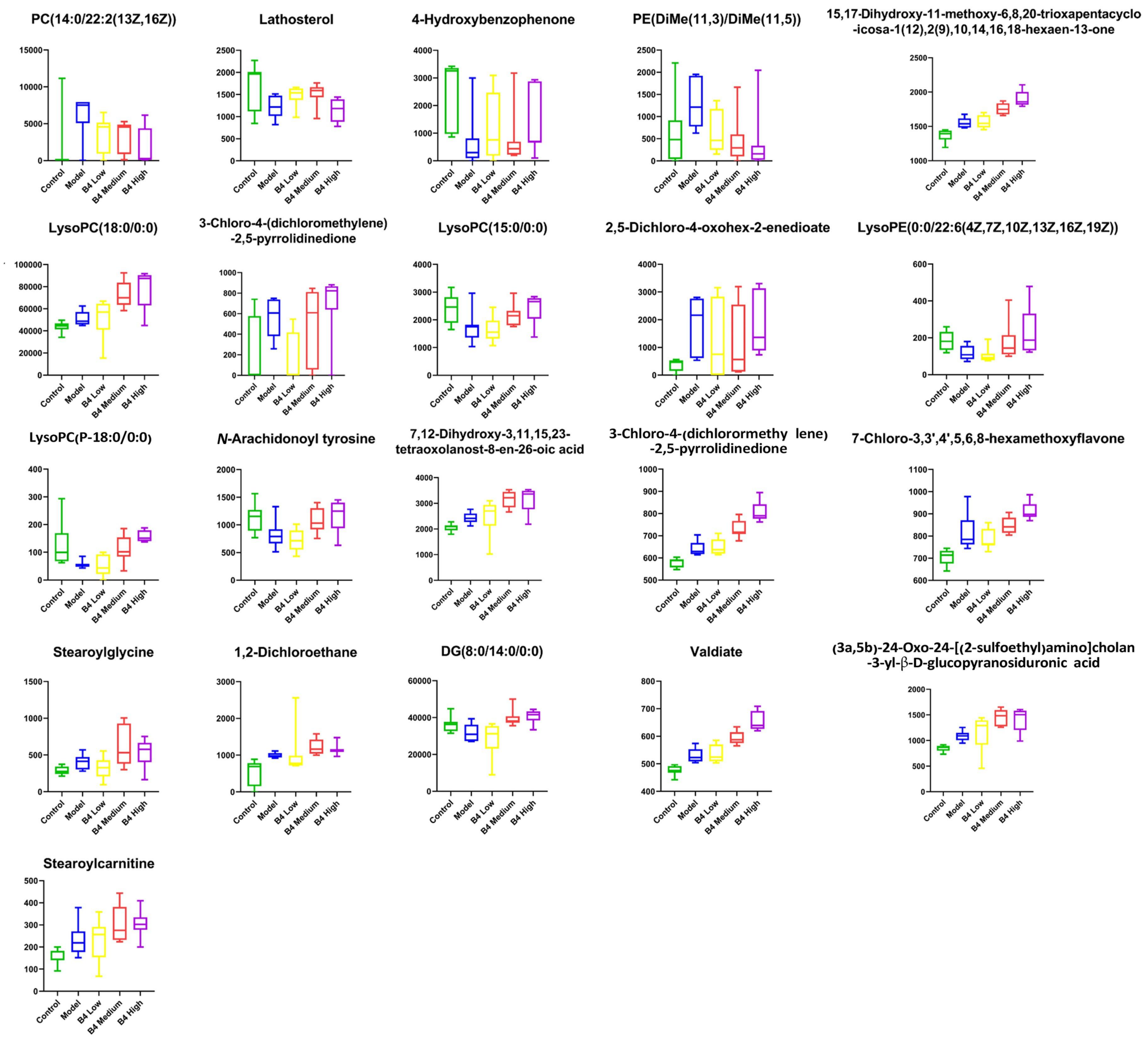

Fig. S2-3. Serum metabonomics study of rat oral ulcer model. G: Box graph to study the regulation of each group of biomarkers after administration.

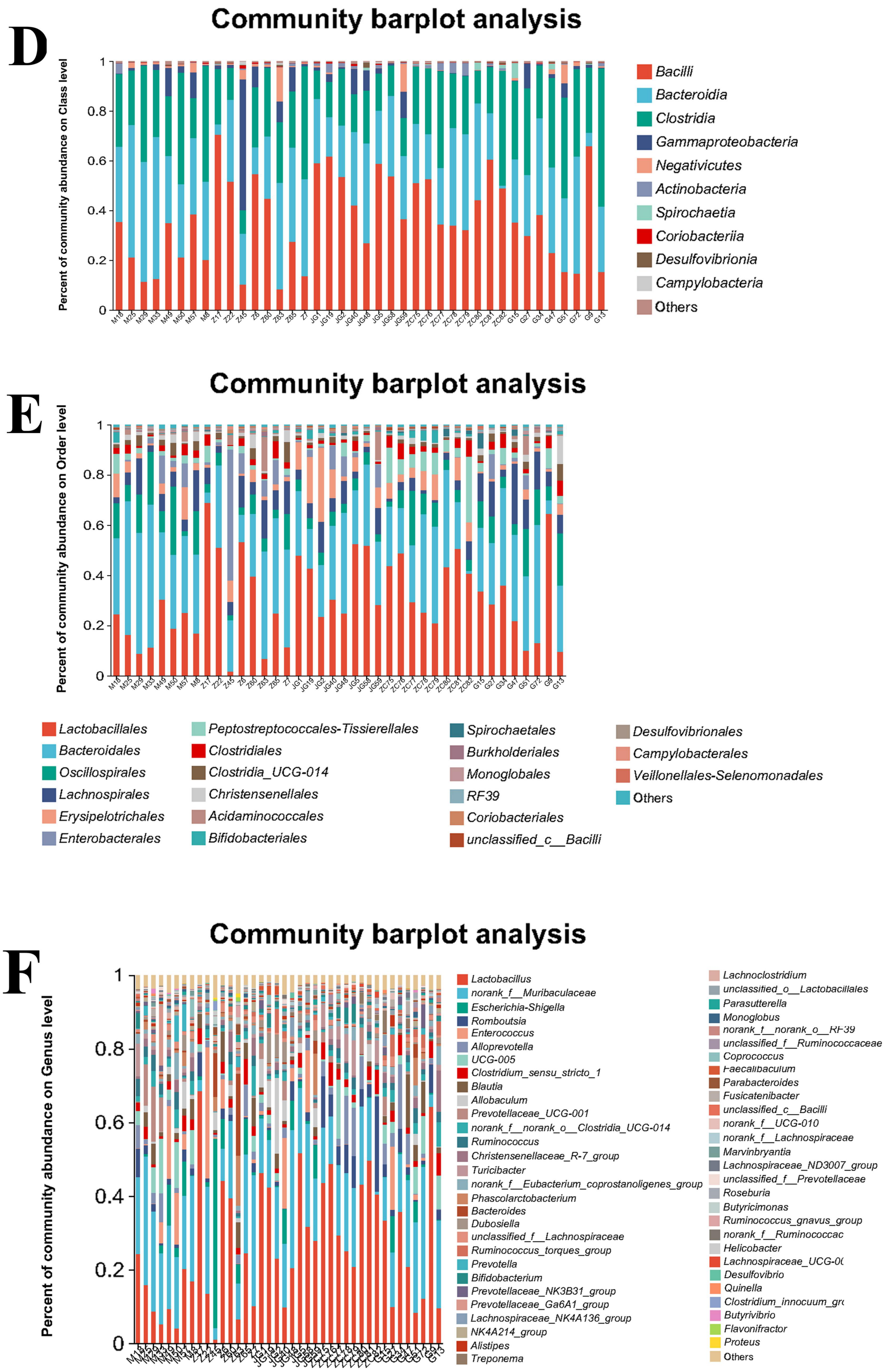

Fig. S3-1. Evaluation of the overall sample of rat intestinal microbiota.D-F: Bacteria at the level of phylum, family, class, order, and genus analysis of the overall species abundance of the population.

G

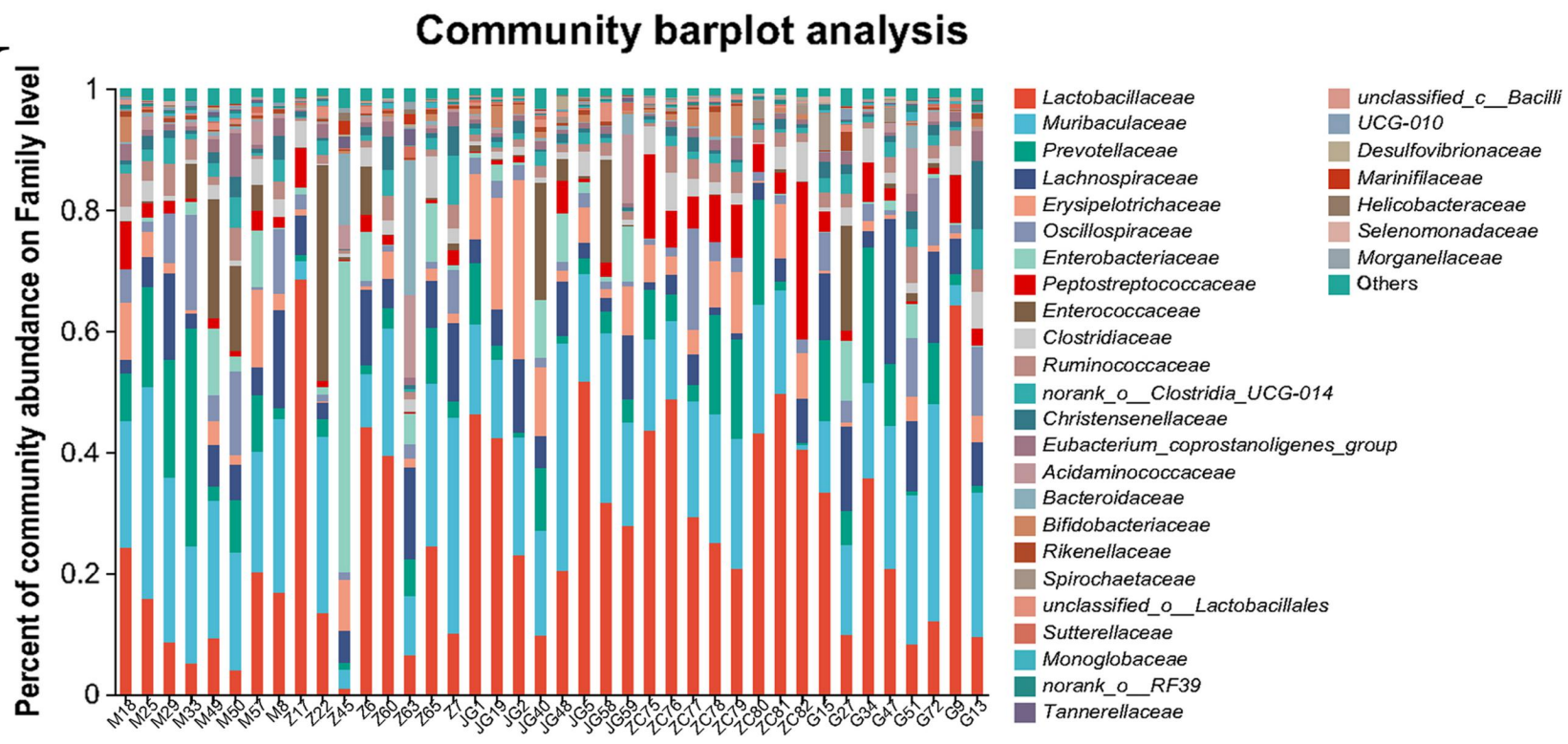
Community barplot analysis
Percent of community abundance on Family level
0
0.2
0.4
0.6
0.8
1
Lactobacillaceae
Muribaculaceae
Prevotellaceae
Lachnospiraceae
Erysipelotrichaceae
Oscillospiraceae
Enterobacteriaceae
Peptostreptococcaceae
Enterococcaceae
Clostridiaceae
Ruminococcaceae
norank_o__Clostridia_UCG-014
Christensenellaceae
Eubacterium_coprostanoligenes_group
Acidaminococcaceae
Bacteroidaceae
Bifidobacteriaceae
Rikenellaceae
Spirochaetaceae
unclassified_o__Lactobacillales
Sutterellaceae
Monoglobaceae
norank_o__RF39
Tannerellaceae
unclassified_c__Bacilli
UCG-010
Desulfovibrionaceae
Marinifilaceae
Helicobacteraceae
Selenomonadaceae
Morganellaceae
Others

H

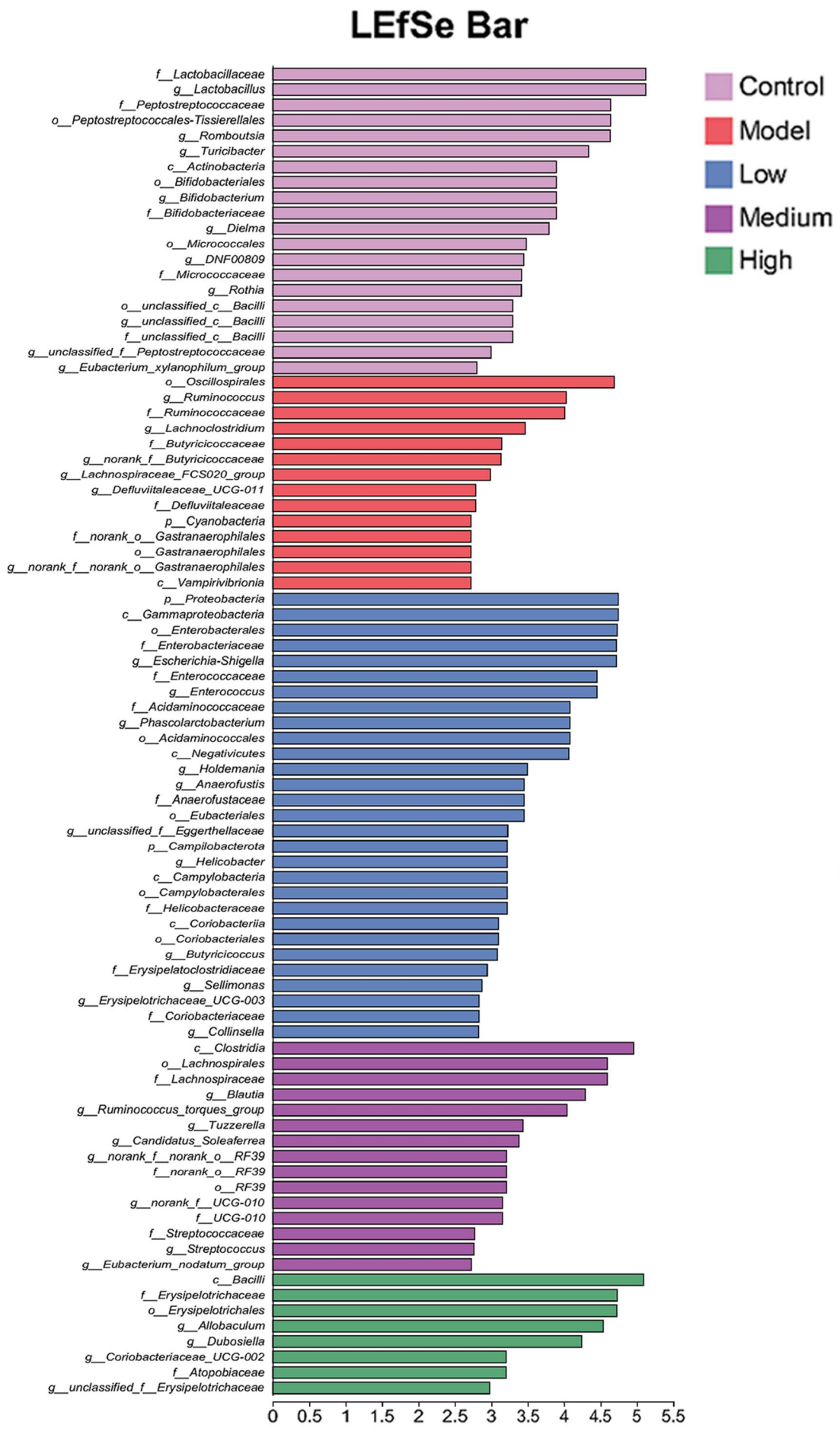
LEfSe Bar
Control
Model
Low
Medium
High
f__Lactobacillaceae
g__Lactobacillus
f__Peptostreptococcaceae
o__Peptostreptococcales-Tissierellales
g__Romboutsia
g__Turicibacter
c__Actinobacteria
o__Bifidobacteriales
g__Bifidobacterium
f__Bifidobacteriaceae
g__Dielma
o__Micrococcales
g__DNF00809
f__Micrococcaceae
g__Rothia
o__unclassified_c__Bacilli
g__unclassified_c__Bacilli
f__unclassified_c__Bacilli
g__unclassified_f__Peptostreptococcaceae
g__Eubacterium_xylanophilum_group
o__Oscillospirales
g__Ruminococcus
f__Ruminococcaceae
g__Lachnoclostridium
f__Butyricicoccaceae
g__norank_f__Butyricicoccaceae
g__Lachnospiraceae_FCS020_group
g__Defluviitaleaceae_UCG-011
f__Defluviitaleaceae
p__Cyanobacteria
f__norank_o__Gastranaerophilales
o__Gastranaerophilales
g__norank_f__norank_o__Gastranaerophilales
c__Vampirivibrionia
p__Proteobacteria
c__Gammaproteobacteria
o__Enterobacterales
f__Enterobacteriaceae
g__Escherichia-Shigella
f__Enterococcaceae
g__Enterococcus
f__Acidaminococcaceae
g__Phascolarctobacterium
o__Acidaminococcales
c__Negativicutes
g__Holdemania
g__Anaerofustis
f__Anaerofustaceae
o__Eubacteriales
g__unclassified_f__Eggerthellaceae
p__Campilobacterota
g__Helicobacter
c__Campylobacteria
o__Campylobacterales
f__Helicobacteraceae
c__Coriobacteriia
o__Coriobacteriales
g__Butyricicoccus
f__Erysipelatoclostridiaceae
g__Sellimonas
g__Erysipelotrichaceae_UCG-003
f__Coriobacteriaceae
g__Collinsella
c__Clostridia
o__Lachnospirales
f__Lachnospiraceae
g__Blautia
g__Ruminococcus_torques_group
g__Tuzzerella
g__Candidatus_Soleaferrea
g__norank_f__norank_o__RF39
f__norank_o__RF39
o__RF39
g__norank_f__UCG-010
f__UCG-010
f__Streptococcaceae
g__Streptococcus
g__Eubacterium_nodatum_group
c__Bacilli
f__Erysipelotrichaceae
o__Erysipelotrichales
g__Allobaculum
g__Dubosiella
g__Coriobacteriaceae_UCG-002
f__Atopobiaceae
g__unclassified_f__Erysipelotrichaceae
0
0.5
1
1.5
2
2.5
3
3.5
4
4.5
5
5.5
LDA SCORE(log10)

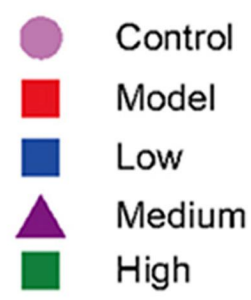
Control
Model
Low
Medium
High

I

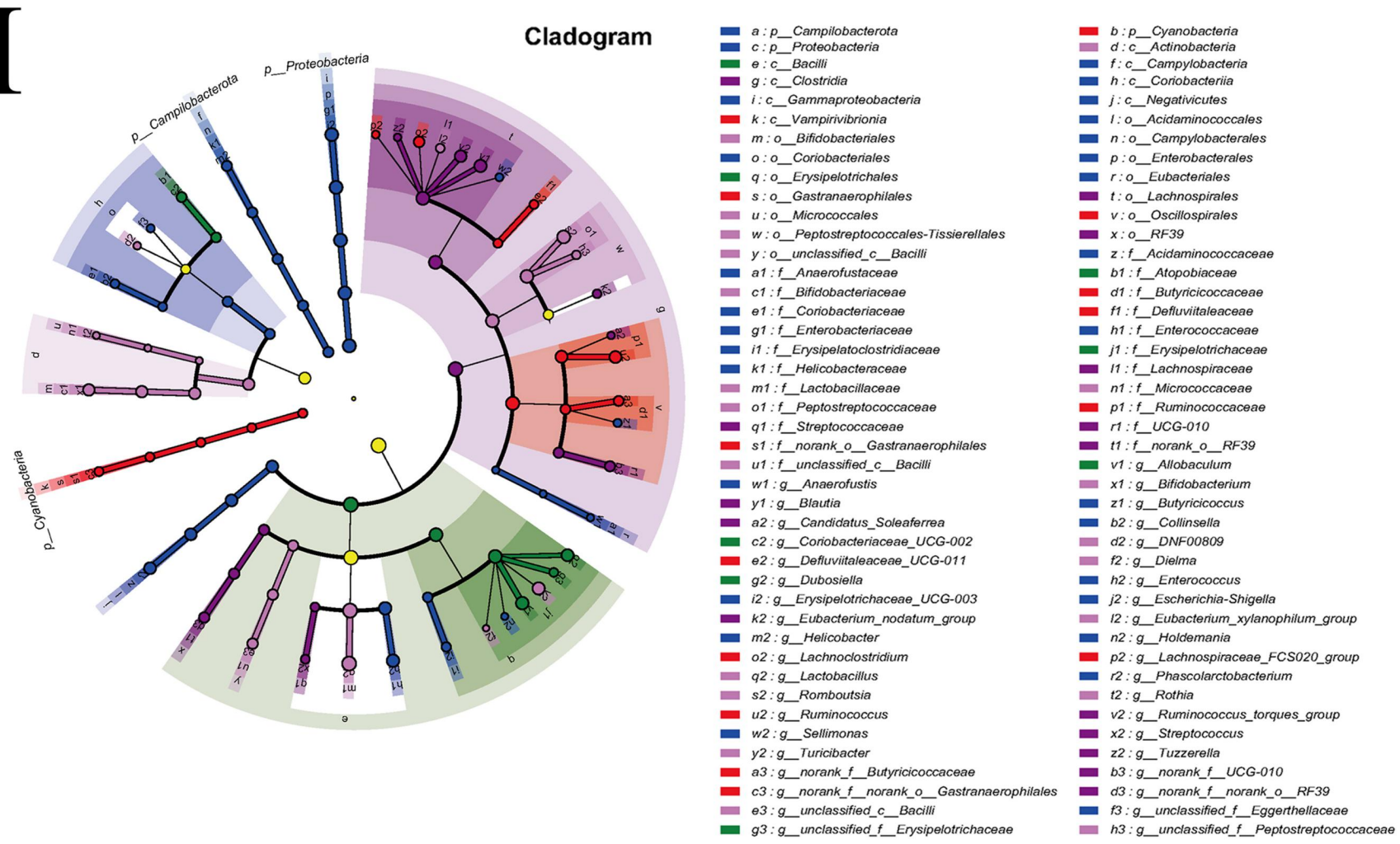
Cladogram
p__Proteobacteria
p__Campilobacterota
p__Cyanobacteria
a : p__Campilobacterota
b : p__Cyanobacteria
c : p__Proteobacteria
d : c__Actinobacteria
e : c__Bacilli
f : c__Campylobacteria
g : c__Clostridia
h : c__Coriobacteriia
i : c__Gammaproteobacteria
j : c__Negativicutes
k : c__Vampirivibrionia
l : o__Acidaminococcales
m : o__Bifidobacteriales
n : o__Campylobacterales
o : o__Coriobacteriales
p : o__Enterobacterales
q : o__Erysipelotrichales
r : o__Eubacteriales
s : o__Gastranaerophilales
t : o__Lachnospirales
u : o__Micrococcales
v : o__Oscillospirales
w : o__Peptostreptococcales-Tissierellales
x : o__RF39
y : o__unclassified_c__Bacilli
z : f__Acidaminococcaceae
a1 : f__Anaerofustaceae
b1 : f__Atopobiaceae
c1 : f__Bifidobacteriaceae
d1 : f__Butyricicoccaceae
e1 : f__Coriobacteriaceae
f1 : f__Defluviitaleaceae
g1 : f__Enterobacteriaceae
h1 : f__Enterococcaceae
i1 : f__Erysipelatoclostridiaceae
j1 : f__Erysipelotrichaceae
k1 : f__Helicobacteraceae
l1 : f__Lachnospiraceae
m1 : f__Lactobacillaceae
n1 : f__Micrococcaceae
o1 : f__Peptostreptococcaceae
p1 : f__Ruminococcaceae
q1 : f__Streptococcaceae
r1 : f__UCG-010
s1 : f__norank_o__Gastranaerophilales
t1 : f__norank_o__RF39
u1 : f__unclassified_c__Bacilli
v1 : g__Allobaculum
w1 : g__Anaerofustis
x1 : g__Bifidobacterium
y1 : g__Blautia
z1 : g__Butyricicoccus
a2 : g__Candidatus_Soleaferrea
b2 : g__Collinsella
c2 : g__Coriobacteriaceae_UCG-002
d2 : g__DNF00809
e2 : g__Defluviitaleaceae_UCG-011
f2 : g__Dielma
g2 : g__Dubosiella
h2 : g__Enterococcus
i2 : g__Erysipelotrichaceae_UCG-003
j2 : g__Escherichia-Shigella
k2 : g__Eubacterium_nodatum_group
l2 : g__Eubacterium_xylanophilum_group
m2 : g__Helicobacter
n2 : g__Holdemania
o2 : g__Lachnoclostridium
p2 : g__Lachnospiraceae_FCS020_group
q2 : g__Lactobacillus
r2 : g__Phascolarctobacterium
s2 : g__Romboutsia
t2 : g__Rothia
u2 : g__Ruminococcus
v2 : g__Ruminococcus_torques_group
w2 : g__Sellimonas
x2 : g__Streptococcus
y2 : g__Turicibacter
z2 : g__Tuzzerella
a3 : g__norank_f__Butyricicoccaceae
b3 : g__norank_f__UCG-010
c3 : g__norank_f__norank_o__Gastranaerophilales
d3 : g__norank_f__norank_o__RF39
e3 : g__unclassified_c__Bacilli
f3 : g__unclassified_f__Eggerthellaceae
g3 : g__unclassified_f__Erysipelotrichaceae
h3 : g__unclassified_f__Peptostreptococcaceae

Fig. S3-2. Evaluation of the overall sample of rat intestinal microbiota. G: Intestinal bacteria at the level of phylum, family, class, order, and genus analysis of the overall species abundance of the population; H: LEfSe analysis of the effect of species abundance on the difference effect; I: Cladogram diagram to explain the significant difference dominant species.

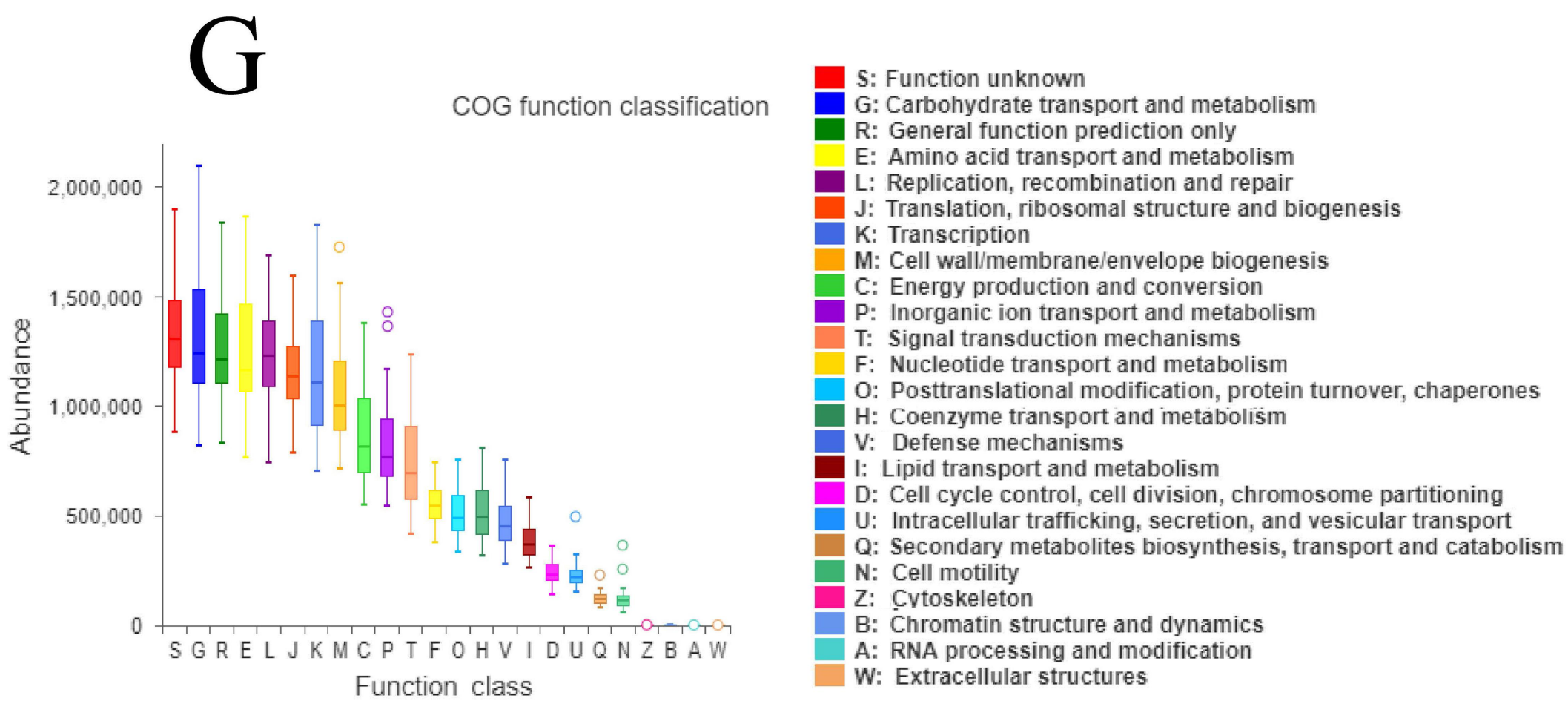

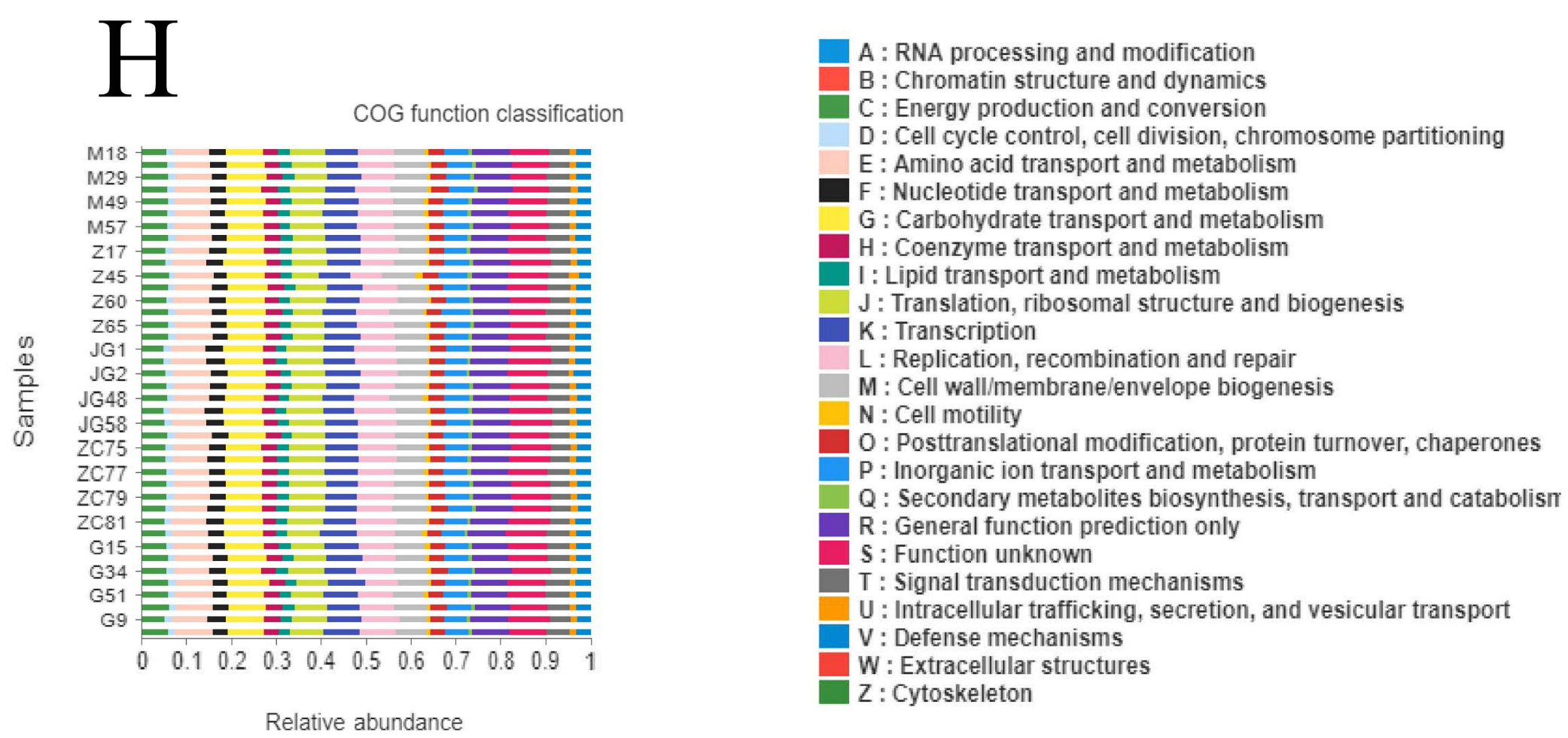

Fig. S4. Microbiota data analysis and function classification. G-H: COG function classification.

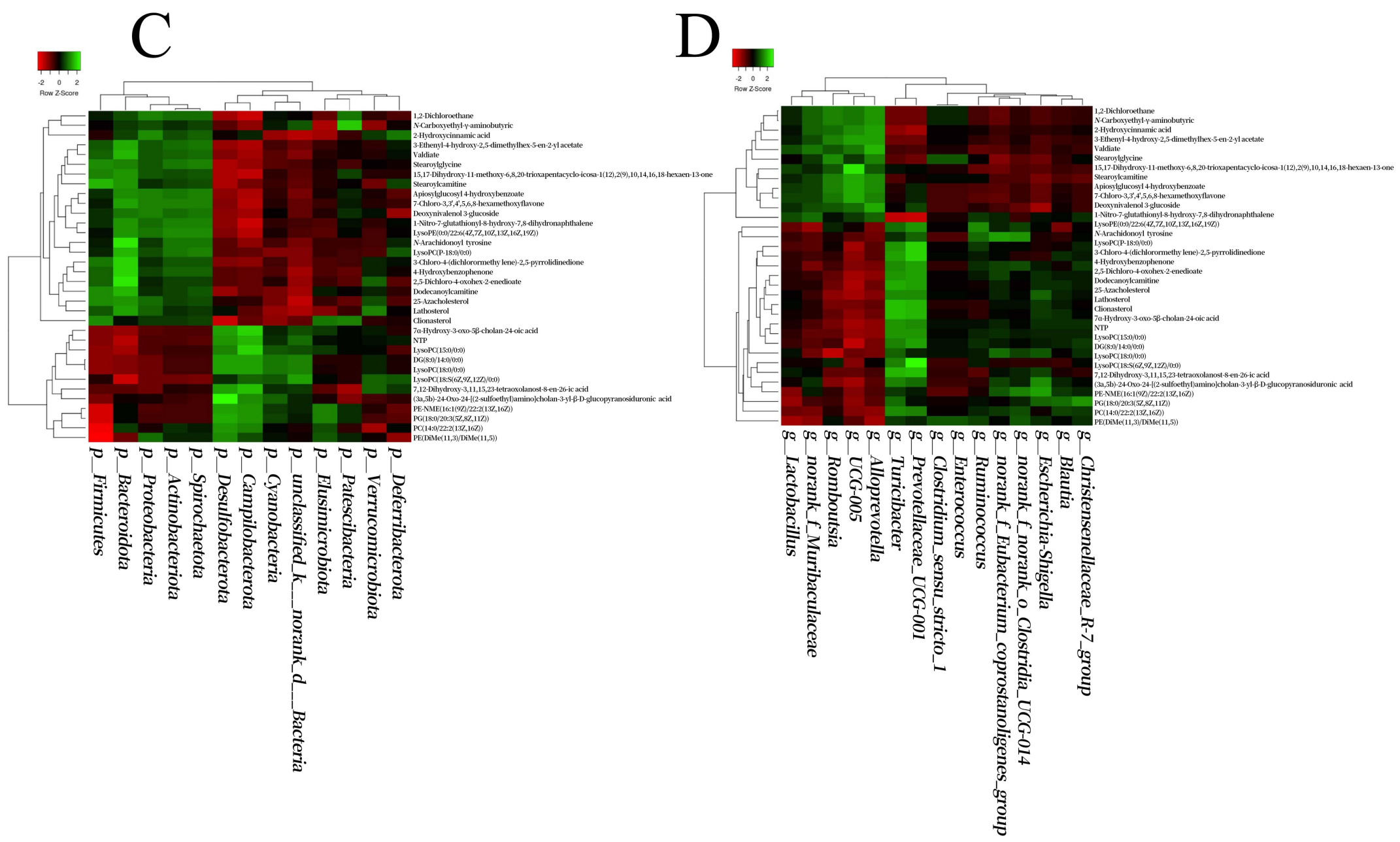

Fig. S5. Analysis of the correlation between biomarkers and intestinal microbiota. C: The difference between the phylum level and the serum biomarker analysis of the association of substances; D: Analysis of the association between different bacterial groups and serum biomarkers at the genus level.

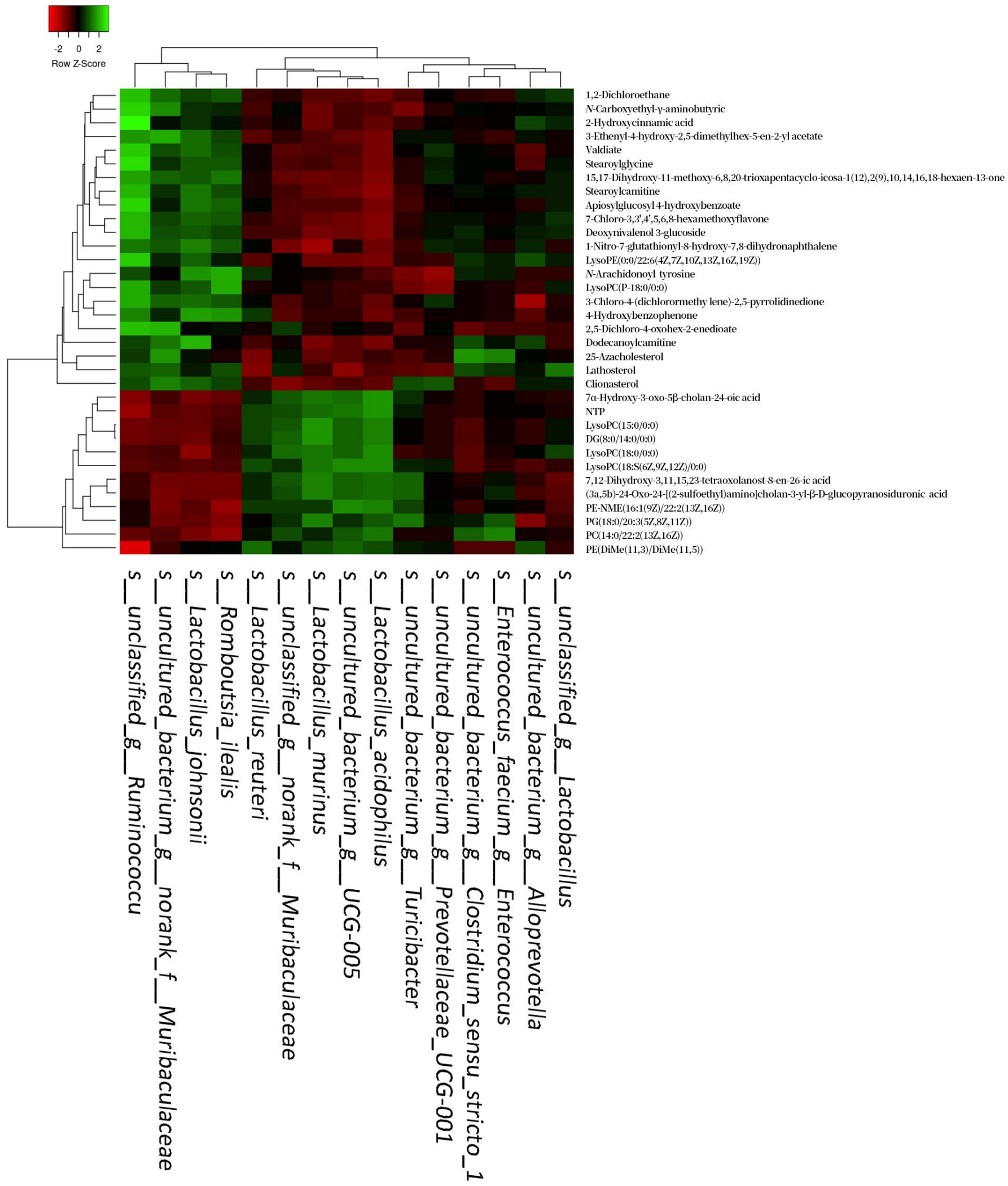

Fig. S6. Gut microbiota analysis. Species level callback
